# Comparison of Nicotine and Toxicant Exposure in Users of Electronic Cigarettes and Combustible Cigarettes

**DOI:** 10.1001/jamanetworkopen.2018.5937

**Published:** 2018-12-14

**Authors:** Maciej L. Goniewicz, Danielle M. Smith, Kathryn C. Edwards, Benjamin C. Blount, Kathleen L. Caldwell, Jun Feng, Lanqing Wang, Carol Christensen, Bridget Ambrose, Nicolette Borek, Dana van Bemmel, Karen Konkel, Gladys Erives, Cassandra A. Stanton, Elizabeth Lambert, Heather L. Kimmel, Dorothy Hatsukami, Stephen S. Hecht, Raymond S. Niaura, Mark Travers, Charles Lawrence, Andrew J. Hyland

**Affiliations:** 1 Department of Health Behavior, Roswell Park Comprehensive Cancer Center, Buffalo, New York; 2 Westat, Rockville, Maryland; 3 Tobacco and Volatiles Branch, Division of Laboratory Sciences, Centers for Disease Control and Prevention, Atlanta, Georgia; 4 Office of Science, Center for Tobacco Products, Food and Drug Administration, Silver Spring, Maryland; 5 National Institute of Drug Abuse, Bethesda, Maryland; 6 Masonic Cancer Center, University of Minnesota, Minneapolis; 7 The Schroeder Institute for Tobacco Research and Policy Studies, Truth Initiative, Washington, DC

## Abstract

**Importance:**

Use of electronic cigarettes (e-cigarettes) is increasing. Measures of exposure to known tobacco-related toxicants among e-cigarette users will inform potential health risks to individual product users.

**Objectives:**

To estimate concentrations of tobacco-related toxicants among e-cigarette users and compare these biomarker concentrations with those observed in combustible cigarette users, dual users, and never tobacco users.

**Design, Setting, and Participants:**

A population-based, longitudinal cohort study was conducted in the United States in 2013-2014. Cross-sectional analysis was performed between November 4, 2016, and October 5, 2017, of biomarkers of exposure to tobacco-related toxicants collected by the Population Assessment of Tobacco and Health Study. Participants included adults who provided a urine sample and data on tobacco use (N = 5105).

**Exposures:**

The primary exposure was tobacco use, including current exclusive e-cigarette users (n = 247), current exclusive cigarette smokers (n = 2411), and users of both products (dual users) (n = 792) compared with never tobacco users (n = 1655).

**Main Outcomes and Measures:**

Geometric mean concentrations of 50 individual biomarkers from 5 major classes of tobacco product constituents were measured: nicotine, tobacco-specific nitrosamines (TSNAs), metals, polycyclic aromatic hydrocarbons (PAHs), and volatile organic compounds (VOCs).

**Results:**

Of the 5105 participants, most were aged 35 to 54 years (weighted percentage, 38%; 95% CI, 35%-40%), women (60%; 95% CI, 59%-62%), and non-Hispanic white (61%; 95% CI, 58%-64%). Compared with exclusive e-cigarette users, never users had 19% to 81% significantly lower concentrations of biomarkers of exposure to nicotine, TSNAs, some metals (eg, cadmium and lead), and some VOCs (including acrylonitrile). Exclusive e-cigarette users showed 10% to 98% significantly lower concentrations of biomarkers of exposure, including TSNAs, PAHs, most VOCs, and nicotine, compared with exclusive cigarette smokers; concentrations were comparable for metals and 3 VOCs. Exclusive cigarette users showed 10% to 36% lower concentrations of several biomarkers than dual users. Frequency of cigarette use among dual users was positively correlated with nicotine and toxicant exposure.

**Conclusions and Relevance:**

Exclusive use of e-cigarettes appears to result in measurable exposure to known tobacco-related toxicants, generally at lower levels than cigarette smoking. Toxicant exposure is greatest among dual users, and frequency of combustible cigarette use is positively correlated with tobacco toxicant concentration. These findings provide evidence that using combusted tobacco cigarettes alone or in combination with e-cigarettes is associated with higher concentrations of potentially harmful tobacco constituents in comparison with using e-cigarettes alone.

## Key Points

**Question:**

Are electronic cigarette (e-cigarette) users exposed to known tobacco-related toxicants and, if so, how does the exposure compare with that of combusted tobacco cigarettes?

**Findings:**

In this population-based cohort study of 5105 participants, current exclusive e-cigarette users had greater concentrations of biomarkers of nicotine, tobacco-specific nitrosamines, volatile organic compounds, and metals compared with never tobacco users. However, these concentrations were lower than those observed in current exclusive cigarette smokers and dual users of both products.

**Meaning:**

Use of e-cigarettes appears to be associated with exposure to known tobacco-related toxicants, but the exposure is reduced compared with cigarette smoking.

## Teaser

This population-based cohort study compares the concentrations of biomarkers of nicotine and other toxicants of cigarettes and electronic cigarettes (e-cigarettes) in smokers who used one or both products vs individuals who have never used tobacco products.

## Introduction

Most cigarette smokers continue to smoke owing to the addictive nature of nicotine.^1^ Cigarettes are harmful nicotine delivery products, exposing smokers to more than 6000 chemicals, many of which are toxic to human health.^1^ Reducing smoking-related health risks requires complete cessation. Yet, among continuing smokers who cannot or will not quit, questions remain about the potential harm of alternative tobacco products, such as electronic cigarettes (e-cigarettes).^2,3^ Approximately 6.7% of US adults have used e-cigarettes within the past 30 days, which translates to roughly 16.1 million Americans.^4^ Most adult e-cigarette users are current or former cigarette smokers (84%),^5,6,7^ and nearly 23% of current multiple tobacco product users report concurrent use of combusted tobacco cigarettes and e-cigarettes (dual users).^4^

Biomarker studies can demonstrate internal exposure to toxic chemicals associated with tobacco product use. Short-term observational studies have shown reduction in biomarker concentrations for volatile organic compounds (VOCs), tobacco-specific nitrosamines (TSNAs), and polycyclic aromatic hydrocarbons (PAHs) in cigarette smokers who switched to e-cigarettes.^8,9,10,11,12^ While tobacco-specific biomarker analyses are useful for interim assessments of exposure, there are several sources of variation to consider when interpreting such data. These include frequency and intensity of tobacco use product type, interindividual and intraindividual variability, biomarker/chemical half-life, and variability in laboratory methods.^13^ This study compared biomarkers of exposure to nicotine and other known tobacco-related toxicants among 4 current tobacco user groups: exclusive users of e-cigarettes (e-cigarette–only users), exclusive users of combusted tobacco cigarettes (cigarette-only smokers), dual users of combusted tobacco cigarettes and e-cigarettes (dual users), and never tobacco product users (reference group).

## Methods

### Data Source

Data are from Wave 1 of the Population Assessment of Tobacco and Health (PATH) Study (2013-2014), a nationally representative, longitudinal cohort study designed to assess tobacco use and health among never, current, or recent former (<12 months) tobacco product users in the US noninstitutionalized adult civilian population.^14^ A detailed overview of design, methods, interview procedures, questionnaires, sampling, weighting, and information on accessing the data are available elsewhere.^14,15^ The weighted response rate for the household screener was 54.0%; among screened households, the weighted Wave 1 adult interview response rate for those providing a urine sample was 63.6%.^14,16^

Westat’s Institutional Review Board approved the study design and protocol. Participants provided written informed consent prior to participation and were compensated for their time.^15^ This study followed the Strengthening the Reporting of Observational Studies in Epidemiology (STROBE) reporting guideline.

Data are from the Biomarker Restricted-Use Files,^16^ which include data from adults (aged ≥18 years) recruited at Wave 1 who agreed to provide a urine sample for analysis. Of those who provided a urine sample, a stratified probability sample of 11 522 adults was selected for laboratory analysis to ensure that respondents represented diverse tobacco product use patterns, including users of multiple tobacco products and never users of any tobacco product; details are provided elsewhere.^16^

A flowchart detailing inclusion and exclusion criteria can be viewed in the eFigure in the Supplement. The analysis consisted of current product users, all of whom reported (1) current everyday or some-days use of cigarettes, e-cigarettes, or both products; (2) no current (everyday or some-days use) use of any other tobacco products; and (3) no use of nicotine replacement therapies in the past 3 days. In addition, cigarette-only smokers and dual users had to report smoking at least 100 cigarettes in their lifetime to be included. We compared current cigarette and e-cigarette users with never users who reported no lifetime tobacco use. Among the 11 522 participants who provided a urine sample, we excluded 6325 who did not meet the product use inclusion criteria, and an additional 92 persons because creatinine level values were outside of the reference range (≤10 mg/dL or >370 mg/dL [to convert to micromoles per liter, multiply by 88.4]).^17,18^

The final analytic sample size was 5105 participants. Sample sizes for each group and class of biomarker (except VOCs) were cigarette-only smokers (n = 2411), e-cigarette–only users (n = 247), dual users (n = 792), and never users of any tobacco products (n = 1655). For VOC metabolites, sample sizes were 2322, 220, 769, and 1571, respectively. This difference was due to a small proportion of samples in each group not being included in the same analytical batch (5%, 11%, 3%, and 4%, respectively).

### Biospecimen Collection and Laboratory Procedures

Detailed biospecimen collection procedures used by the PATH Study are described elsewhere.^15,16^ Participants self-collected full-void spot urine samples in 500-mL polypropylene containers. Samples were immediately placed in a Crēdo Cube shipper, which transported samples between 2°C and 8°C, and were shipped overnight to the study biorepository for storage and processing. Biomarkers were subsequently measured using highly selective mass spectrometric methods at the Centers for Disease Control and Prevention’s Division of Laboratory Sciences.^19,20,21,22,23,24,25,26,27,28,29,30^ A complete list of measured biomarkers and references to analytical methods and limits of detection (LOD) are provided in eTable 1 in the Supplement.

### Outcomes

This study examined 50 biomarkers associated with exposure to tobacco. Biomarkers were selected from several classes of known tobacco product constituents, including (1) nicotine metabolites, (2) TSNAs, (3) metals, (4) PAHs, and (5) VOCs. A complete listing of geometric means for all 50 biomarkers among each user group can be found in eTable 2 in the Supplement.

A representative biomarker from each panel was selected for visualization of study results based on its documented association with tobacco exposure and linkage to tobacco-related disease development or adverse health effects. These biomarkers included metabolites of nicotine, tobacco-specific nitrosamine 4-(methylnitrosamino)-1-(3-pyridyl)-1-butanone (NNK), lead, cadmium, naphthalene, pyrene, acrylonitrile, acrolein, and acrylamide.^13,31,32,33,34,35,36,37,38,39,40,41,42^ To estimate nicotine exposure, total nicotine equivalents were calculated by taking the molar sum of cotinine and trans-3′-hydroxycotinine values. Table 1 outlines the potential clinical relevance of selected biomarkers.

**Table 1.** Known or Suspected Diseases or Disorders Related to Exposure to Selected Toxic Chemicals Measured in the Study

Group	Chemical Exposure	Internal Biomarker	Clinical Relevance to Known or Suspected Diseases or Disorders	FDA HPHC in Tobacco Product Classification^11^
Urinary nicotine metabolites	Nicotine	TNE2^a^	Linked to impaired brain and lung development (fetal stage), altered development of cerebral cortex and hippocampus (adolescent stage), causal factor in nicotine dependence, addiction, and withdrawal disorders^33^	RDT, AD
Tobacco-specific nitrosamines	4-(Methylnitros- amino)-1-(3-pyridyl)- 1-butanone	4-(Methylnitrosamino)- 1-(3-pyridyl)- 1-butanol	Known human carcinogen (International Agency for Research on Cancer Group 1)^39^	CA
Metals	Lead	Urinary lead	Associated with brain damage, mental retardation, behavioral problems, developmental delays (fetal/youth/adolescent stage); linked to damage of sense organs and nerves, impaired cognitive function, as well as hearing and vision impairment in adults^37^	CA, CT, RDT
	Cadmium	Urinary cadmium	International Agency for Research on Cancer Group 1 known human carcinogen with adverse effects on kidney and bone; implicated in risk of diseases that involve tissues and organ systems where cadmium accumulates, including eye tissues^38^	CA, RT, RDT
Polycyclic aromatic hydrocarbons	Naphthalene	2-Napthol	International Agency for Research on Cancer group 2B possible human carcinogen; linked to respiratory tract lesions, including tumors in the upper respiratory tract demonstrated in animal studies, and respiratory irritation in humans^31^	CA
				RT
	Pyrene	1-Hydroxypyrene	Accepted biomarker of carcinogenic polycyclic aromatic hydrocarbons; associated with lipid damage and alterations of endogenous and exogenous antioxidants; contributes to oxidative stress, associated with acute myocardial infarction^35^	Not included
Volatile organic compounds	Acrylonitrile	*N*-Acetyl-S- (2-cyanoethyl)- l-cysteine	International Agency for Research on Cancer group 2B carcinogen^32^	CA
				RT
	Acrolein	*N*-Acetyl-S- (2-carboxyethyl)- l-cysteine	Vapor form may cause eye, nasal, and respiratory tract irritations in low-level exposure^34,42^	RT
				CT
	Acrylamide	*N*-Acetyl-*S*- (2-carbamoylethyl)- l-cysteine	Probable contributor to cancer risk and an International Agency for Research on Cancer group 2A carcinogen^32^; acrylamide can be neurotoxic in humans and animals, causes peripheral neuropathies in workers who use or work in the manufacture of the product, and is a reproductive toxicant in mice^40^	CA

Abbreviations: AD, addictive; CA, carcinogen; CT, cardiovascular toxicant; FDA, US Food and Drug Administration; HPHC, harmful or potentially harmful constituent; RDT, reproductive or developmental toxicant; RT, respiratory toxicant; TNE2, total nicotine equivalents.

^a^ TNE2 calculated by taking molar sum of urinary cotinine and trans-3′-hydroxycotinine values.

### Statistical Analysis

Statistical analyses were performed between November 4, 2016, and October 5, 2017. Descriptive analyses were conducted to compare demographic characteristics between tobacco user groups, including Pearson χ^2^ tests and 1-way analysis of variance. Geometric mean concentrations of each biomarker were compared between tobacco user groups. Biomarker values below the assay LOD were imputed using a common substitution formula (LOD/√2).^43^ Biomarker variables for which more than 40% of the observations were below the LOD are flagged in the tables owing to uncertainty or instability of the estimates. Data for each biomarker were right skewed; all were transformed using the natural log. Data were creatinine level corrected to adjust for differences in hydration status on sample collection.

We excluded fewer than 4.6% of the observations for any single biomarker owing to interfering substances in laboratory assays. We performed sensitivity analyses to determine what, if any, association was present between these exclusions and our results. Analyses showed that excluding these observations did not significantly alter the results. The results from laboratory analysis for all other observations are reported for each biomarker; proportions above the LOD listed in tables are reflective of the listed sample sizes for each biomarker and user group. All analyses were weighted using PATH Study sample weights and the probability of selection for laboratory analyses (eMethods in the Supplement).

Variance estimation was approached using balanced, repeated replications with the Fay adjustment set to 0.3 to enhance the precision of the estimates. The 95% CIs were computed using a log scale and were transformed to reflect geometric mean values for the upper and lower bounds. Estimates with relative SEs greater than 30% were flagged owing to estimate stability concerns.

Multivariable linear regression models were constructed to calculate geometric means and 95% CIs were determined to estimate differences in non–creatinine level–adjusted biomarker concentrations outcomes by tobacco user group. All models were constructed using a forward stepwise approach; covariates associated with tobacco user group status with *P* < .20 and covariables that were considered biologically and statistically relevant were included in the final models (urinary creatinine level, age, sex, self-reported race/ethnicity, and educational level). Missing data on demographic covariates were imputed as described in the PATH Study Restricted-Use Files User Guide. To control for other potential sources of smoke exposure, models were also adjusted for self-reported past 30-day use of marijuana (yes or no), as well as for self-reported number of hours per week exposed to secondhand smoke (SHS) at work, home, school, or outdoors. Frequency of product use was classified based on self-reported every-day use or some-days use of cigarettes and/or e-cigarettes.

Exploratory analyses were conducted among dual users to examine whether frequency of dual use was associated with concentration of nicotine and toxicants. A description of statistical models examining differences in biomarkers among dual users is provided in eMethods in the Supplement.

All analyses were completed using *svy* commands in Stata, version 14.0.^44^ All tests were 2-tailed and adjusted for multiple comparisons using the Bonferroni method. *P* values <.05 were considered statistically significant.

## Results

Of the 5105 participants, most were aged 35 to 54 years (weighted percentage, 38%; 95% CI, 35%-40%), women (60%; 95% CI, 59%-62%), and non-Hispanic white (61%; 95% CI, 58%-64%). Nearly all (93% [229]) e-cigarette–only users previously smoked cigarettes. The e-cigarette–only users who were former established smokers (had smoked ≥100 cigarettes in their lifetime and do not currently smoke) quit smoking a mean (SD) of 3.5 (9.0) years before sample collection, and they previously smoked a mean of 19.0 cigarettes per day (CPD) (95% CI, 16.9-21.1). Among all e-cigarette–only users, 56% (147) used e-cigarettes daily, and 44% (100) used e-cigarettes on some days. Among dual users, 20% (145) used e-cigarettes daily (95% CI, 16.3%-24.6%), while 80% (647) used them on some days (95% CI, 75.3%-83.7%). Cigarette consumption was similar between cigarette-only smokers and dual users (dual users, 15.1 CPD [95% CI, 14.1-16.0 CPD] vs cigarette-only, 15.4 CPD [95% CI, 14.1-16.6 CPD], *t* = 0.34, *P* = .73) (Table 2). These 2 groups also had similar SHS exposure. Table 2 displays demographic characteristics of the sample. The proportion of users with detectable concentrations of each biomarker are reported in eTable 2 in the Supplement.

**Table 2.** Participant Demographics, Current Everyday/Some-Day e-Cigarette–only Users, Dual Users, Cigarette Smokers, and Never Users, Population Assessment of Tobacco and Health Wave 1 (2013-2014) (N = 5105)^a^^,^^b^

Characteristic	No. (%) [95% CI, %]			
	Never Users (n = 1655)	e-Cigarette–Only Users (n = 247)	Dual Users (n = 792)	Cigarette-Only Smokers (n = 2411)
Sex				
Men	607 (37) [35-40]	98 (41) [33-49]	283 (37) [33-42]	1059 (46) [43-49]
Women	1048 (63) [60-65]	149 (59) [51-67]	509 (63) [58-67]	1352 (54) [51-57]
Age, y				
18-24	679 (16) [14-18]	54 (17) [13-22]	116 (9) [7-11]	376 (9) [8-11]
25-34	243 (17) [15-20]	68 (29) [22-36]	189 (23) [20-26]	556 (22) [20-25]
35-54	419 (36) [33-40]	80 (32) [25-40]	337 (45) [41-49]	970 (42) [38-45]
≥55	314 (31) [28-34]	45 (22) [17-28]^c^	150 (24) [20-28]	509 (27) [24-30]
Race/ethnicity				
White, non-Hispanic	785 (56) [52-60]	180 (73) [67-79]	607 (81) [78-84]	1601 (69) [66-73]
Nonwhite, non-Hispanic	467 (23) [21-26]	42 (17) [13-24]^c^	100 (10) [8-12]	494 (19) [16-22]
Hispanic	403 (21) [18-24]	25 (9) [6-13]^c^	85 (9) [7-11]	316 (12) [9-15]
Educational level				
Less than HS/GED	290 (16) [14-19]	46 (21) [16-27]^a^	190 (22) [19-25]	719 (28) [25-31]
HS graduate	433 (25) [22-29]	65 (28) [22-34]	166 (22) [19-26]	610 (30) [27-33]
Some college/associate degree	567 (27) [24-31]	102 (39) [32-45]	345 (42) [37-47]	852 (32) [29-36]
≥Bachelor degree	365 (31) [27-35]	34 (13) [9-18]^c^	91 (14) [11-18]	230 (10) [8-12]
Past 30-d marijuana use, % yes	20 (0.59) [0.35-0.99]^c^	40 (15) [10-22]^c^	122 (13) [11-16]	425 (16) [14-19]
Everyday use, %				
Cigarettes	NA	NA	650 (83) [79-86]	1975 (4) [81-87]
e-Cigarettes	NA	147 (56) [49-63]	145 (20) [16-25]	NA
Use nicotine-containing e-cigarettes, %	NA	161 (59) [51-65]	306 (38) [34-42]	NA
Use flavored e-cigarettes, %	NA	122 (44) [37-52]	208 (25) [21-30]	NA
SHS exposure, mean (95% CI), h/wk^d^	2.0 (1.5-2.4)	7.9 (5.7-10.1)	20.0 (17.5-22.5)	18.9 (16.7-21.2)
e-Cigarettes per day, mean, (95% CI)^e^	NA	1.3 (0.9-1.7)	0.6 (0.3-0.9)	NA
Cigarettes per day, mean (95% CI)	NA	NA	15.1 (14.1-16.0)	15.4 (14.1-16.6)

Abbreviations: GED, general education development; HS, high school; NA, not applicable; SHS, secondhand smoke.

^a^ Statistically significant differences were detected in examining tobacco use status by sex, age, race/ethnicity, educational level, daily cigarette use, daily e-cigarette use, use of nicotine-containing e-cigarettes, use of flavored e-cigarettes, and past 30-day marijuana use (Pearson χ^2^ test, *P* < .05). Mean CPD did not differ significantly between dual users and cigarette-only smokers; e-cigarettes/d was significantly different between e-cigarette–only users and dual users (independent samples *t* test, *P* < .05). Exposure to SHS significantly varied based on tobacco user status (1-way analysis of variance, *P* < .05). Percentages may not total to 100% owing to rounding.

^b^ All percentages are weighted. Never users answered no to lifetime use of any tobacco product; e-cigarette–only users reported yes to current everyday/some-days use of e-cigarettes, no to current everyday/some-days use of all other tobacco products; dual users reported yes to current everyday/some-days use of tobacco cigarettes and e-cigarettes, no to current everyday/some-days use of all other tobacco products; cigarette-only smokers reported yes to current everyday/some-days use of cigarettes, no to current everyday/some-days use of all other tobacco products. All user groups reported no past 3-day use of nicotine replacement therapies.

^c^ Flagging due to denominator less than 50.

^d^ Measured according to the following question: “During the past 7 days, about how many hours were you around others who were smoking, [whether or not you were smoking yourself]? Include time in your home, in a car, at work, or outdoors.”

^e^ Measured according to the following questions: everyday users, “On average, about how many [e-cigarettes/e-cigarettes cartridges] do you now use each day?”; someday/experimental users, “On how many of the past 30 days did you use an e-cigarette?” and “On average, on those [number of days/days you used], how many [e-cigarettes/e-cigarettes cartridges] did you usually use each day?” Measures were combined into a composite variable to assess e-cigarette use frequency across groups.

### Never Users vs e-Cigarette–Only Users

**Figure 1.**
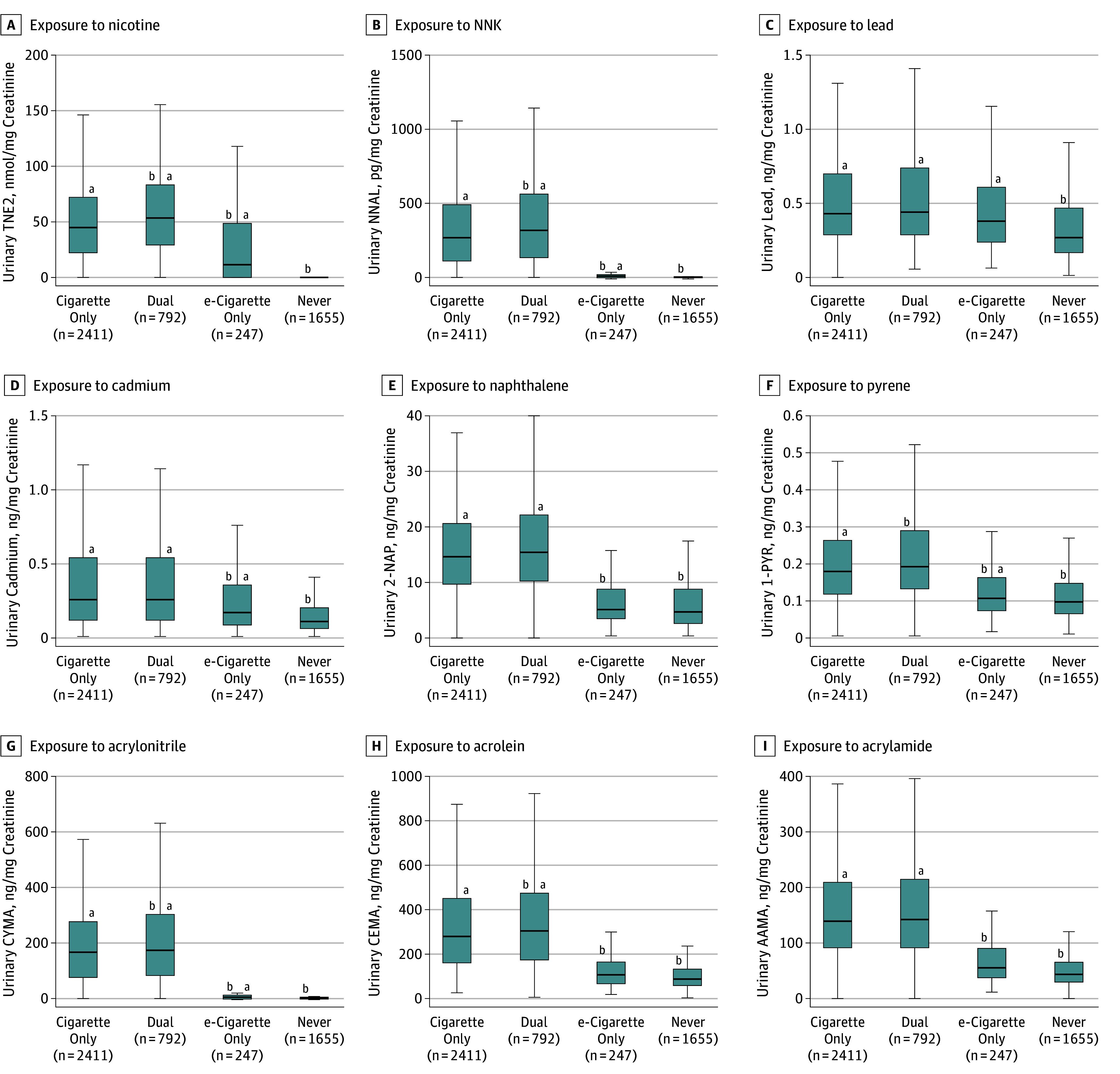
Biomarkers of Exposure Among Dual Users, Cigarette-Only Smokers, e-Cigarette–Only Users, and Never Users, Population Assessment of Tobacco and Health Study, Wave 1, 2013-2014 (N = 5105) Exposure to nicotine (TNE2) (A), tobacco-specific nitrosamine 4-(methylnitrosamino)-1-(3-pyridyl)-1-butanone (NNK) (B), lead (C), cadmium (D), naphthalene (2-naphthol) (2-NAP) (E), pyrene (1-hydroxypyrene) (1-PYR) (F), acrylonitrile (CYMA) (G), acrolein (CEMA) (H), and acrylamide (AAMA) (I). All analyses are weighted. Some volatile organic compound laboratory results were outstanding at the time these analyses were conducted, so weighted estimates may not accurately reflect values in the target population. Box depicts median (interquartile range); whiskers depict minimum and maximum values for creatinine level–corrected biomarker values across tobacco user groups. Outliers excluded from figure to facilitate presentation clarity. ^a^Statistically significant difference from never users adjusted for urinary creatinine level, cigarettes per day, age, sex, race/ethnicity, educational level, secondhand smoke exposure, past 30-day marijuana use, and tobacco use status. ^b^Statistically significant difference cigarette-only users adjusted for urinary creatinine level, cigarettes per day, age, sex, race/ethnicity, educational level, secondhand smoke exposure, past 30-day marijuana use.

The distribution of selected biomarkers among never users, e-cigarette–only users, cigarette-only smokers, and dual users are presented in Figure 1. Compared with e-cigarette–only users, never users had significantly lower geometric mean concentrations of all major nicotine metabolites and total nicotine equivalents, all TSNAs, 4 metals, 1 PAH, and 4 VOCs. Compared with e-cigarette–only users, geometric mean concentrations of NNAL were approximately 81% lower in never users (creatinine-adjusted geometric mean for never users: 0.921; 95% CI, 0.819-1.035 pg/mg creatinine; e-cigarette–only users: 4.887; 95% CI, 3.817-6.257 pg/mg creatinine, effect size after adjustment: *t* = 7.73, *P* < .001), and the concentration of biomarkers of metal exposure including lead (never users: 0.351; 95% CI, 0.330-0.373 ng/mg creatinine; e-cigarette–only users: 0.432; 95% CI, 0.382-0.488 ng/mg creatinine; *t* = 3.71, *P* < .001) and cadmium (never users: 0.149; 95% CI, 0.140-0.159 ng/mg creatinine; e-cigarette–only users: 0.193; 95% CI, 0.165-0.225 ng/mg creatinine, *t* = 6.60, *P* < .001) were approximately 19% and 23% lower in never users, respectively. Never users exhibited significantly lower concentrations of the biomarker for pyrene (never users: 0.128; 95% CI, 0.121-0.136 ng/mg creatinine; e-cigarette–only users: 0.161; 95% CI, 0.143-181 ng/mg creatinine, *t* = 2.19, *P* = .03), geometric mean concentrations were approximately 20% lower than e-cigarette–only users. Never users had significantly lower concentrations of the biomarker for acrylonitrile (never users: 1.315; 95% CI, 1.230-1.406 ng/mg creatinine, e-cigarette–only users: 3.959; 95% CI, 3.002-5.219 ng/mg creatinine, *t* = 5.55, *P* < .001), which were approximately 66% lower than e-cigarette–only users (Figure 1).

The frequency of e-cigarette use influenced the differences in observed urinary concentrations of several biomarkers among daily vs some-days e-cigarette users: everyday e-cigarette–only users had higher concentrations of all major nicotine metabolites, 2 TSNAs, 2 metals (lead and strontium) and 1 marker for acrylonitrile (eTable 3 in the Supplement) than some-days e-cigarette–only users.

### e-Cigarette–Only Users vs Cigarette-Only Smokers

**Figure 2.**
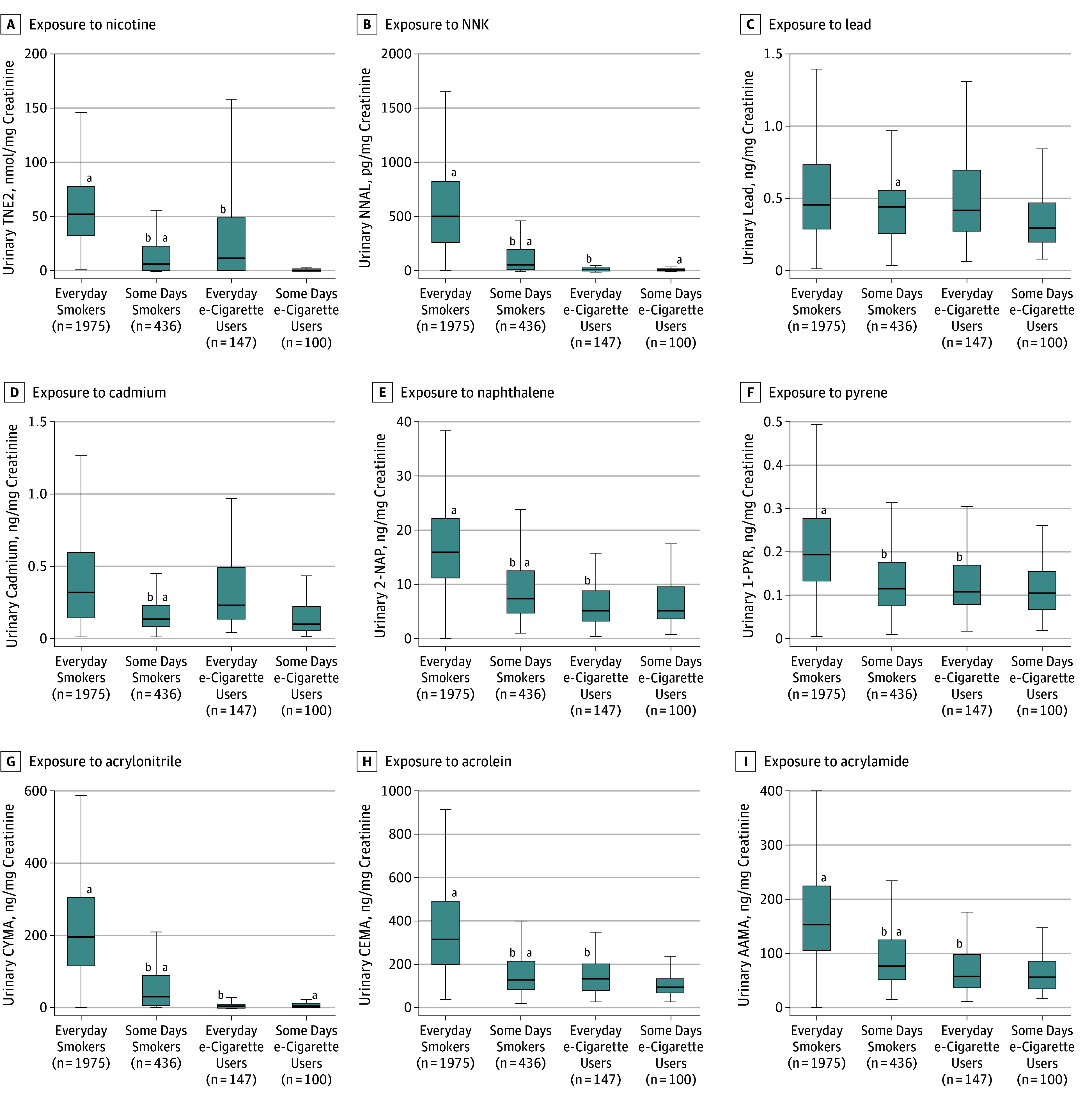
Biomarkers of Exposure Among e-Cigarette–Only Users and Cigarette-Only Smokers, Stratified by Everyday or Some-Days Product Use, Population Assessment of Tobacco and Health Study, Wave 1, 2013-2014 (n = 2658) Exposure to nicotine (TNE2) (A), tobacco-specific nitrosamine 4-(methylnitrosamino)-1-(3-pyridyl)-1-butanone (NNK) (B), lead (C), cadmium (D), naphthalene (2-naphthol) (2-NAP) (E), pyrene (1-hydroxypyrene) (1-PYR) (F), acrylonitrile (CYMA) (G), acrolein (CEMA) (H), and acrylamide (AAMA) (I). All analyses are weighted. Some volatile organic compound laboratory results were outstanding at the time these analyses were conducted, so weighted estimates may not accurately reflect values in the target population. Everyday e-cigarette users reported yes to current daily use of e-cigarettes, no to current everyday/some-day use of all other tobacco products; some-days e-cigarette users reported yes to current some-days use of e-cigarettes, no to current everyday/some-days use of all other tobacco products; everyday smokers reported yes to current daily use of cigarettes, no to current everyday/some-days use of all other tobacco product; some-days smokers reported yes to current some days use of cigarettes, no to current everyday/some-days use of all other tobacco products. All user groups reported no past 3-day use of nicotine replacement therapies. Box depicts median (interquartile range); whiskers depict minimum and maximum values for creatinine-corrected biomarker across tobacco user groups. Outliers excluded from figure to facilitate presentation clarity. ^a^Statistically significant difference with everyday e-cigarette users based on results from adjusted linear regression models (*P* < .05). Comparisons were performed for everyday smokers with everyday e-cigarette users, everyday smokers with some-days smokers, everyday e-cigarette users with some-days smokers, and everyday e-cigarette users with some-days e-cigarette users. ^b^Statistically significant difference with everyday smokers based on results from adjusted linear regression models (*P* < .05).

The e-cigarette–only users and cigarette-only smokers were found to have statistically similar biomarker concentrations of nearly all metals (except cadmium) and 3 VOCs (markers for toluene, benzene, and carbon disulfide). Compared with cigarette-only smokers, e-cigarette–only users were found to have significantly lower concentrations of all major nicotine metabolites, 2 minor tobacco alkaloids, all TSNAs, 1 metal (cadmium), all PAHs, and 17 VOCs. Geometric mean concentrations of total nicotine equivalents (e-cigarette only users: 2.000; 95% CI, 1.100-3.500 nmol/mg creatinine; cigarette-only smokers: 27.90, 95% CI, 23.80-32.70 nmol/mg creatinine; *t* = 8.53, *P* < .001) and NNAL (e-cigarette–only users: 4.887; 95% CI, 3.817-6.257 pg/mg creatinine; cigarette-only smokers: 203.5; 95% CI, 181.7-227.9 pg/mg creatinine; *t* = 27.96, *P* < .001) were approximately 93% and 98% lower in e-cigarette–only users compared with cigarette-only smokers, respectively. Cadmium concentrations were 30% lower in e-cigarette–only users compared with cigarette-only smokers (e-cigarette–only users: 0.193; 95% CI, 0.165-0.225 ng/mg creatinine; cigarette-only smokers: 0.277; 95% CI, 0.259-0.297 ng/mg creatinine; *t* = 2.40, *P* = .02). Among the PAH biomarkers, naphthalene was approximately 62% lower (e-cigarette–only users: 5.287; 95% CI, 4.693-5.956 ng/mg creatinine, cigarette-only smokers: 13.91; 95% CI, 13.21-14.65 ng/mg creatinine; *t* = 15.00, *P* < .001) and pyrene was 47% lower (e-cigarette–only users: 0.161; 95% CI, 0.143-0.181 ng/mg creatinine; cigarette-only smokers: 0.303; 95% CI, 0.287-0.321 ng/mg creatinine; *t* = 10.22, *P* < .001) in e-cigarette–only users compared with cigarette-only smokers. The e-cigarette–only users also exhibited significantly lower concentrations of VOCs, with a 60% decrease in concentrations of the biomarker for acrolein (e-cigarette–only users: 108.0; 95% CI, 95.93-121.6 ng/mg creatinine; cigarette-only smokers: 271.5; 95% CI, 255.1-289.0 ng/mg creatinine; *t* = 13.26, *P* < .001), 97% decrease in concentrations of acrylonitrile (e-cigarette–only users: 3.959; 95% CI, 3.002-5.219 ng/mg creatinine; cigarette-only smokers: 123.9; 95% CI, 109.9-139.7 ng/mg creatinine; *t* = 23.65, *P* < .001), and 59% decrease in concentrations of the biomarker for acrylamide (e-cigarette–only users: 56.05; 95% CI, 51.07-61.50 ng/mg creatinine; cigarette-only smokers: 136.4; 95% CI, 129.3-143.8 ng/mg creatinine; *t* = 17.53, *P* < .001). A similar pattern of findings emerged when evaluating everyday and some-days users separately (Figure 2).

### Dual Users vs Cigarette-Only Smokers

Compared with all cigarette-only smokers, all dual users had significantly higher concentrations of most biomarkers, including most major nicotine metabolites, 3 TSNAs (including NNAL), 2 metals, 5 PAHs (including the biomarker for pyrene), and 13 VOCs (including biomarkers for acrolein and acrylonitrile). Cigarette-only smokers exhibited biomarker concentrations that were approximately 36% lower for total nicotine equivalents (dual users: 43.70; 95% CI, 39.80-48.10 nmol/mg creatinine; cigarette-only smokers: 27.90; 95% CI, 23.80-32.70 nmol/mg creatinine; *t* = 4.78, *P* < .001); and 23% lower for NNAL (dual users: 262.6; 95% CI, 240.0-287.3 pg/mg; cigarette-only smokers: 203.5; 95% CI, 181.7-227.9 pg/mg creatinine; *t* = 4.39, *P* < .001) than dual users. Geometric mean concentrations of lead and cadmium did not differ between dual users and cigarette-only smokers (0.500; 95% CI, 0.475-0.526 ng/mg creatinine vs 0.479; 95% CI, 0.462-0.496 ng/mg creatinine, *t* = 1.66, *P* = .10 and 0.280; 95% CI, 0.256-0.305 ng/mg creatinine vs 0.277; 95% CI, 0.259-0.297 ng/mg creatinine; *t* = 0.11, *P* = .91, respectively). The PAH and VOC concentrations were slightly lower in cigarette-only smokers compared with dual users, with 15% lower geometric mean concentrations for pyrene (dual users: 0.355; 95% CI, 0.339-0.373 ng/mg creatinine; cigarette-only smokers: 0.303; 95% CI, 0.287-0.321 ng/mg creatinine; *t* = 3.63, *P* < .001), 10% lower geometric means for acrolein (dual users: 302.0; 95% CI, 283.3-321.8 ng/mg creatinine; cigarette-only smokers: 271.5; 95% CI, 255.1-289.0 ng/mg creatinine; *t* = 2.38, *P* = .02), and 15% lower geometric mean concentrations for acrylonitrile (dual users: 146.2; 95% CI, 133.8-159.8 ng/mg creatinine; cigarette-only smokers: 123.9; 95% CI, 109.9-139.7 ng/mg creatinine; *t* = 2.42, *P* = .02). There were no statistically significant differences in other biomarker concentrations between dual users and cigarette-only smokers.

**Figure 3.**
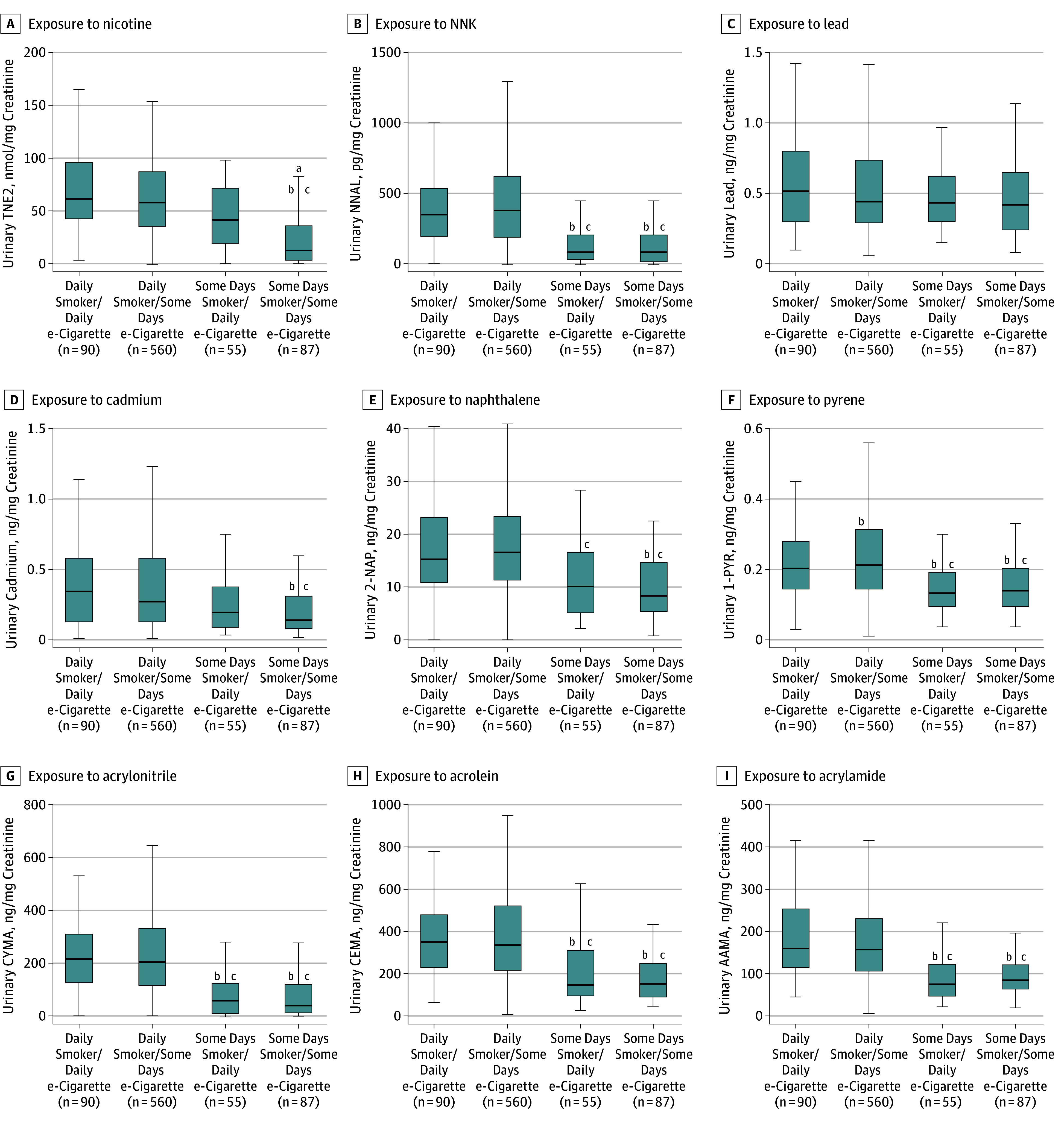
Biomarkers of Exposure Among Dual Users of Tobacco Cigarettes and e-Cigarettes, Population Assessment of Tobacco and Health Study, Wave 1, 2013-2014 (n = 792) Exposure to nicotine (TNE2) (A), tobacco-specific nitrosamine 4-(methylnitrosamino)-1-(3-pyridyl)-1-butanone (NNK) (B), lead (C), cadmium (D), naphthalene (2-naphthol) (2-NAP) (E), pyrene (1-hydroxypyrene) (1-PYR) (F), acrylonitrile (CYMA) (G), acrolein (CEMA) (H), and acrylamide (AAMA) (I). All analyses are weighted. Some volatile organic compound laboratory results were outstanding at the time these analyses were conducted, so weighted estimates may not accurately reflect values in the target population. Mean number of cigarettes per day (CPD) in everyday smokers/everyday e-cigarette users was 15.9 (95% CI 13.8-18.1); some days smokers/everyday e-cigarette users, 14.3 CPD (on days smoked) (95% CI 4.6-24.0 CPD); everyday smokers/some-days e-cigarette users, 16.2 CPD (95% CI, 15.5-16.9); and some-days smokers/some-days e-cigarette users, 6.1 CPD (on days smoked) (95% CI, 4.2-8.0 CPD). Box depicts median and interquartile range; whiskers depict minimum and maximum values for creatinine level–corrected biomarker values across tobacco user groups. Outliers excluded from figure to facilitate presentation clarity. ^a^Denotes estimate with relative SE greater than 30%. ^b^Statistically significant difference from everyday smokers/some days e-cigarette users adjusted for urinary creatinine level, CPD, age, sex, race/ethnicity, educational level, secondhand smoke exposure, and past 30-day marijuana use. ^c^Statistically significant difference from everyday smokers/everyday e-cigarette users adjusted for urinary creatinine level, CPD, age, sex, race/ethnicity, educational level, secondhand smoke exposure, past 30-day marijuana use, and tobacco use status.

Figure 3 displays the distribution of selected biomarkers among dual users per frequency of product use. Dual users who used cigarettes everyday but differed in frequency of e-cigarette use (everyday or some-days use) had the same cigarette consumption: everyday smokers/everyday e-cigarette users had a mean of 15.9 CPD (95% CI, 13.8-18.1 CPD), and everyday smokers/some-days e-cigarette users had a mean of 16.2 CPD (95% CI, 15.5-16.9 CPD). On the days they smoked cigarettes, some-days smokers/everyday e-cigarette users had a mean of 14.3 CPD (95% CI, 4.6-24.0 CPD), while some-days smokers/some-days e-cigarette users had a mean of 6.1 CPD (95% CI, 4.2-8.0 CPD).

Findings suggest everyday smokers/everyday e-cigarette users had significantly higher concentrations of all major nicotine metabolites, 2 minor tobacco alkaloids, all PAHs (including naphthalene), and 15 VOCs (including biomarkers for acrylamide and acrylonitrile) compared with some-days smokers/some-days e-cigarette users (eTable 4 in the Supplement). Everyday smokers/everyday e-cigarette users had significantly higher concentrations of all TSNAs (including NNAL), 5 PAHs (including the biomarker for pyrene), and 10 VOCs (including biomarkers for acrylamide and acrylonitrile) compared with some-days smokers/everyday e-cigarette users. Except for 6 biomarkers, everyday smokers/everyday e-cigarette users were statistically similar to everyday smokers/some-days e-cigarette users. Compared with some-days smokers/everyday e-cigarette users, everyday smokers/some-days e-cigarette users had higher concentrations of all TSNAs (including NNAL), all PAHs, and 17 VOCs.

## Discussion

Findings suggest exclusive e-cigarette use results in measurable exposure to tobacco-related constituents; however, compared with cigarette smoking, biomarker concentrations of nicotine and toxicants among e-cigarette–only users were much lower. Dual users exhibited higher concentrations of exposure to nearly all biomarkers compared with cigarette-only smokers. Further investigation can identify contributing factors resulting in higher toxicant concentrations among dual users. Continued daily consumption of tobacco cigarettes among dual users may play a role, as 82% of dual users reported daily cigarette smoking. Biomarker concentrations were positively correlated with cigarette smoking frequency.

Several biomarkers measured in this study are metabolites of known carcinogens as well as respiratory, cardiovascular, and/or reproductive/developmental toxicants.^36^ Although this study does not evaluate the association between exposure and disease among exclusive e-cigarette users or dual users, data clearly show that e-cigarette users are exposed to known tobacco-related toxicants. The degree to which such e-cigarette-related toxicant exposures affect disease incidence merits further research. Thus, users of e-cigarettes should be aware that these products are sources of exposure to toxicants that are linked to illness, but the degree to which e-cigarette use may facilitate or hinder the development of disease downstream remains unknown.

These findings suggest lower concentrations of biomarkers of nicotine exposure among e-cigarette–only users compared with cigarette-only smokers. The timeframe covered by this analysis corresponds to the increasing availability of first-generation e-cigarette products.^5^ Early-generation devices are reported to be inefficient nicotine delivery systems, which our findings support.^45^ Advances in later-generation device technology that contribute to nicotine and toxicant exposure (ie, improved nicotine delivery, adjustable power and temperature settings) suggest that the results presented in this article related to exposures from exclusive e-cigarette use may be conservative. For instance, use of later-generation e-cigarettes with variable temperature controls has been linked to greater yields of carbonyl compounds, including acrolein (measured in the present study), formaldehyde, and acetaldehyde (for which no validated urinary biomarkers of exposure exist).^46,47^ However, a study examining nicotine and toxicant concentrations in a convenience sample of long-term exclusive users of primarily later-generation e-cigarettes showed similar nicotine delivery and lower toxicant exposure to exclusive cigarette smokers.^12^ Because users’ experience with specific devices, individual user puff topography, and device characteristics all interact with respect to nicotine and toxicant exposure,^47^ further research is needed in more recent markets that reflect widespread newer-generation e-cigarette use.

The present study also found little variability in concentrations of metals between user groups. Metals have long half-lives (ie, years) and may come from sources other than tobacco exposure; thus, biomarker concentrations may reflect exposures from prior use of tobacco products and cigarette smoking (not necessarily e-cigarette use).^13^ As expected, several biomarkers associated with combustion byproducts (eg, PAHs, VOCs) were significantly higher among cigarette-only smokers. Detectable concentrations of TSNAs can also result from passive smoke exposure^13^; e-cigarette–only users had significantly greater passive smoke exposure than never users. This difference might reflect circumstances under which e-cigarette users may be differentially exposed (eg, having more friends who smoke conventional cigarettes); however, our analysis was able to control for SHS exposure.

The PATH Study provides the research community with a unique opportunity to examine biomarker data collected from a large, population-based sample representative of never, current, or recent former (<12 months) tobacco product users in the US noninstitutionalized adult civilian population. From these data, we examined 50 biomarkers of exposure for tobacco-related toxicants that are addictive or otherwise potentially harmful to human health.^36^ As far as we are aware, no other study to date has looked at such a wide range of biomarkers to assess exposures by frequency of use among a general population of tobacco users. The large panel of biomarkers examined here reflects one of the most comprehensive examinations of exposures related to e-cigarette use and/or cigarette smoking to date and demonstrates the importance of frequency of product use in resulting biomarker concentrations among e-cigarette and dual users. Other studies, including the National Health and Nutrition Examination Survey, have measured select biomarkers of tobacco exposure and reported similar findings among limited numbers of e-cigarette users. The large sample of e-cigarette users in this study allows for a comprehensive analysis of exposure patterns to numerous chemicals that may have health implications for e-cigarette users.^48,49^

### Limitations

Several limitations should be considered. First, questionnaire data at Wave 1 did not differentiate between use of first-, second-, third-, or later-generation e-cigarette devices by participants. These factors have been demonstrated to be important in differential exposure to nicotine and toxicants among e-cigarette users in other studies.^47^ Readers should interpret several of the estimates presented here with caution, particularly those from exploratory analyses among dual users. For example, the distributions of biomarkers among certain groups of dual users (eg, some-days e-cigarette/some-days cigarette smokers) may be a better indicator of the product last used, which may or may not be e-cigarettes. Biomarker estimates should also be interpreted in the context of compound half-lives listed in eTable 1 in the Supplement. For instance, elevated cadmium concentrations in e-cigarette users could be remnants from prior combusted cigarette use, given the metal’s long half-life and the fact that 93% of exclusive e-cigarette users in our sample are former cigarette smokers.^13^ Given the small number of e-cigarette–only users who never used cigarettes (n = 18), we cannot investigate how smoking history may influence resulting concentrations of biomarkers with longer half-lives. However, most biomarkers examined have a short half-life (mean, 1.5-10 hours).^13^ In addition, some biomarkers (eg, arsenic) lack sensitivity in identifying exposure to tobacco-related constituents; such biomarkers may come from other sources, such as diet or environmental pollution.^13^Also, e-cigarettes may generate unique toxicant exposures (eg, nickel and chromium) or exposures to toxicants not presently designated as harmful or potentially harmful tobacco product constituents, such as those associated with e-cigarette flavorings.^50,51^

## Conclusions

These data suggest that current, exclusive e-cigarette use results in exposure to known toxicants. Toxicant exposure is greatest among dual users, and frequency of use of combustible cigarettes is positively correlated with tobacco product toxicant concentration. These findings provide evidence that using combusted tobacco cigarettes alone or in combination with e-cigarettes is associated with higher concentrations of potentially harmful tobacco constituents in comparison with using e-cigarettes alone. This study may provide a foundation for disease risk investigations in the PATH Study population.

## Notes

Supplement. **eFigure.** Study Inclusion/Exclusion **eTable 1.** Analytical Limits of Detection (LOD), Biological Half-lives, and Assay Methods for Nicotine and Toxicants Assessed at Wave 1, PATH Study, 2013-2014 **eTable 2**. Biomarkers of Exposure to Tobacco-Related Compounds and Other Chemicals, Among Current Never Users, e-Cigarette Only-Users, Dual Users, and Cigarette-Only Smokers (Normalized for Creatinine, Geometric Means, 95% Confidence Intervals), PATH Wave 1 (2013-2014) (n=5,105) **eMethods.** Details on Model Building Procedures for Dual User Analysis and Sample Weighting **eTable 3.** Biomarkers of Exposure to Tobacco-Related Compounds and Other Chemicals, Among Everyday and Someday Users of e-Cigarettes and Cigarettes (Normalized for Creatinine, Geometric Means, 95% Confidence Intervals), PATH Wave 1 (2013-2014) (n=2,658) **eTable 4.** Biomarkers of Exposure to Tobacco-Related Compounds and Other Chemicals, Among Dual Users (Normalized for Creatinine, Geometric Means, 95% Confidence Intervals), PATH Wave 1 (2013-2014) (n=792)
eReferences
