# Supplementary material for: Comparison of Nicotine and Toxicant Exposure in Users of Electronic Cigarettes and Combustible Cigarettes
Source: JAMA Netw Open. 2018 Dec 14;1(8):e185937. doi: 10.1001/jamanetworkopen.2018.5937 (PMC6324349; doi:10.1001/jamanetworkopen.2018.5937)
Supplement: Supplement. — eFigure. Study Inclusion/Exclusion eTable 1. Analytical Limits of Detection (LOD), Biological Half-lives, and Assay Methods for Nicotine and Toxicants Assessed at Wave 1, PATH Study, 2013-2014 eTable 2. Biomarkers of Exposure to Tobacco-Related Compounds and Other Chemicals, Among Current Never Users, e-Cigarette Only-Users, Dual Users, and Cigarette-Only Smokers (Normalized for Creatinine, Geometric Means, 95% Confidence Intervals), PATH Wave 1 (2013-2014) (n=5,105) eMethods. Details on Model Building Procedures for Dual User Analysis and Sample Weighting eTable 3. Biomarkers of Exposure to Tobacco-Related Compounds and Other Chemicals, Among Everyday and Someday Users of e-Cigarettes and Cigarettes (Normalized for Creatinine, Geometric Means, 95% Confidence Intervals), PATH Wave 1 (2013-2014) (n=2,658) eTable 4. Biomarkers of Exposure to Tobacco-Related Compounds and Other Chemicals, Among Dual Users (Normalized for Creatinine, Geometric Means, 95% Confidence Intervals), PATH Wave 1 (2013-2014) (n=792) eReferences [file jamanetwopen-e185937-s001.pdf]

Supplementary Online Content

Goniewicz ML, Smith DM, Edwards KC, et al. Comparison of nicotine and toxicant exposure in users of electronic cigarettes and combustible cigarettes. *JAMA Netw Open*. 2018;1(8):e185937. doi:10.1001/jamanetworkopen.2018.5937

eFigure. Study Inclusion/Exclusion

eTable 1. Analytical Limits of Detection (LOD), Biological Half-lives, and Assay Methods for Nicotine and Toxicants Assessed at Wave 1, PATH Study, 2013-2014

eTable 2. Biomarkers of Exposure to Tobacco-Related Compounds and Other Chemicals, Among Current Never Users, e-Cigarette Only-Users, Dual Users, and Cigarette-Only Smokers (Normalized for Creatinine, Geometric Means, 95% Confidence Intervals), PATH Wave 1 (2013-2014) (n=5,105)

eMethods. Details on Model Building Procedures for Dual User Analysis and Sample Weighting

eTable 3. Biomarkers of Exposure to Tobacco-Related Compounds and Other Chemicals, Among Everyday and Someday Users of e-Cigarettes and Cigarettes (Normalized for Creatinine, Geometric Means, 95% Confidence Intervals), PATH Wave 1 (2013-2014) (n=2,658)

eTable 4. Biomarkers of Exposure to Tobacco-Related Compounds and Other Chemicals, Among Dual Users (Normalized for Creatinine, Geometric Means, 95% Confidence Intervals), PATH Wave 1 (2013-2014) (n=792)

eReferences

This supplementary material has been provided by the authors to give readers additional information about their work.

© 2018 Goniewicz ML et al. *JAMA Network Open.*

eFigure. Study Inclusion/Exclusion

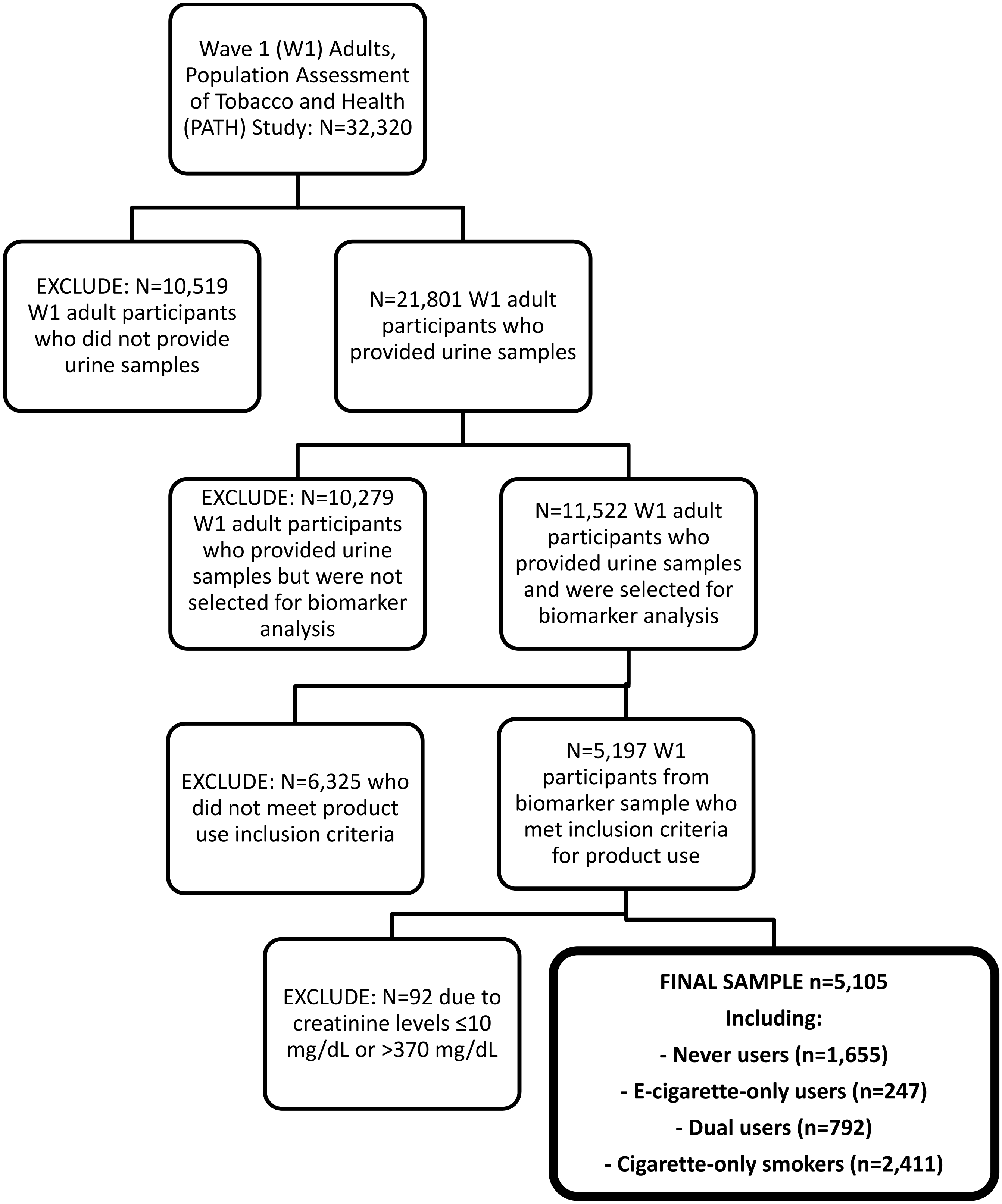

© 2018 Goniewicz ML et al. *JAMA Network Open.*

eTable 1. Analytical Limits of Detection (LOD), Biological Half-lives, and Assay Methods for Nicotine and Toxicants Assessed at Wave 1, PATH Study, 2013-2014

Biomarker	Limit of Detection	Biological Half-lifves	Analytical Assay Method
**Urinary Nicotine Metabolites^[1] (ng/mL)**			
trans-3'-Hydroxycotinine (HCTT)	0.030	6.4 hrs	All nicotine metabolites were assessed using two separate isotope dilution high performance liquid chromatography/ tandem mass spectrometric (HPLC-MS/MS) methods.[2,3]
Cotinine (COTT)	0.030	16-18 hrs	
Nicotine (NICT)	10.5	1-2 hrs	
Cotinine N-oxide (COXT)	2.02	N/A	
Nicotine 1'-oxide (NOXT)	2.50	N/A	
Norcotinine (NCCT)	1.11	N/A	
Nornicotine (NNCT)	2.50	N/A	
**Minor Tobacco Alkaloids (ng/mL)**			
Anabasine (ANBT)	0.51	16 hrs	All minor tobacco alkaloids were assessed using two separate isotope dilution high performance liquid chromatography/tandem mass spectrometric (HPLC-MS/MS) methods.[2,3]
Anatabine (ANTT)	0.39	10 hrs	
**Arsenic and Arsenic Compounds (ug/L)^**			
Arsenous Acid	0.12	10 hrs	All arsenic compounds were assessed using high performance liquid chromatography/inductively coupled plasma dynamic reaction cell mass spectrometry (HPLC-ICP-DRC-MS).[4,5]
Arsenic Acid	0.79	10 hrs	
Dimethylarsinic acid	1.19	10 hrs	
Monomethylarsonic acid	0.20	10 hrs	
**Tobacco Specific Nitrosamines (TSNAs) (ng/L)**			
4-methylnitrosamino)-4-(3-pyridyl)-1-butanol (NNAL)	0.6	10.3 days[6]	All TSNAs were assessed using isotope dilution high performance liquid chromatography/atmospheric pressure chemical ionization tandem mass spectrometry (HPLC-MS/MS).[7]
N'-nitrosonornicotine (NNN)	2.8	N/A	
N'-nitrosoanatabine (NAT)	4.2	N/A	
N'-nitrosoanabasine (NAB)	1.6	N/A	

© 2018 Goniewicz ML et al. *JAMA Network Open.*

Biomarker	Limit of Detection	Biological Half-lifves	Analytical Assay Method
	**Metals (µg/L)**		
Beryllium (UBE)	0.016	Several years	All metals were assessed using inductively coupled plasma mass spectrometry (ICP-MS). [8,9]
Cadmium (UCD)	0.036	13.6 years	
Cobalt (UCO)	0.023	Several days	
Manganese (UMN)	0.13	39 days	
Lead (UPB)	0.03	1-2 months in blood & soft tissues, years to decades in bone	
Strontium (USR)	2.34	47.3 hrs	
Thallium (UTL)	0.018	1-3 days	
Uranium (UUR)	0.002	24 hrs	
	**Polycyclic Aromatic Hydrocarbons[10] (ng/L)**		
1-Naphthol or 1-hydroxynaphthalene (1-NAP)	60	4.3 hrs	All PAHs were assessed using enzymatic hydrolysis, on-line solid phase extraction, and isotope dilution liquid chromatography tandem mass spectrometry.[11]
2-Naphthol or 2-hydroxynaphthalene (2-NAP)	90	9.4 hrs	
3-Hydroxyfluorene (3-FLU)	8	8.2 hrs	
2-Hydroxyfluorene (2-FLU)	8	2.1 hrs	
1-Hydroxyphenanthrene (1-PHE)	9	5.1 hrs	
1-Hydroxypyrene (1-PYR)	70	6.0 hrs	
2-Hydroxyphenanthrene and 3-Hydroxyphenanthrene (2-3PHE)	10	4.1 hrs	
	**Volatile Organic Compounds (VOCs) (ng/mL)**		
2-Methylhippuric acid (2MHA) (Xylene)	5.00	34 hrs	All VOCs were assessed using isotope dilution UPLC-MS/MS.[12,13]
3, 4-Methylhippuric acid (34MH) (Xylene)	8.00	34 hrs	
N-Acetyl-S-(2-carbamoylethyl)-L-cysteine (AAMA) (Acrylamide)	2.20	11 or 17.4 hrs	
N-Acetyl-S-(N-methylcarbamoyl)-L-cysteine (AMCA) (N,N-Dimethylformamide/isocyanates)	6.26	23 hrs	
N-Acetyl-S-(benzyl)-L-cysteine (BMA) (Toluene)	0.50	<10 hrs	
N-Acetyl-S-(2-carboxyethyl)-L-cysteine (CEMA) (Acrolein)	6.96	N/A	
N-Acetyl-S-(1-cyano-2-hydroxyethyl)-L-cysteine (CYHA) (Acrylonitrile)	2.60	N/A	
N-Acetyl-S-(2-cyanoethyl)-L-cysteine (CYMA) (Acrylonitrile)	0.50	8 hrs	
N-Acetyl-S-(3,4-dihydroxybutyl)-L-cysteine (DHBM) (1,3-Butadiene)	5.25	N/A	
N-Acetyl-S-(2-carbamoyl-2-hydroxyethyl)-L-cysteine (GAMA) (Acrylamide)	9.40	19 or 25.1 hrs	
N-Acetyl-S-(2-hydroxyethyl)-L-cysteine (HEMA) (Acrylonitrile, vinyl	0.79	>5 hrs	

© 2018 Goniewicz ML et al. *JAMA Network Open.*

Biomarker	Limit of Detection	Biological Half-lifves	Analytical Assay Method
chloride, ethylene oxide)			
N-Acetyl-S-(2-hydroxypropyl)-L-cysteine (HPM2) (Propylene Oxide)	5.30	N/A	
N-Acetyl-S-(3-hydroxypropyl)-L-cysteine (HPMA) (Acrolein)	13.0	N/A	
N-Acetyl-S-(3-hydroxypropyl-1-methyl)-L-cysteine (HPMM) (Crotonaldehyde)	3.00	N/A	
N-Acetyl-S-(4-hydroxy-2-methyl-2-buten-1-yl)-L-cysteine (IPM3) (Isoprene)	0.74	N/A	
Mandelic acid (MADA)	12.0	2.1,3.6, or 3.9 hrs	
N-Acetyl-S-(4-hydroxy-2-buten-1-yl)-L-cysteine (MHB3) (1,3 Butadiene)	0.60	>9 or <6 hrs*	
Phenylglyoxylic acid (PGHA) (Ethylbenzene, styrene)	12.0	8.1,8.8 or 10.5 hrs	
N-Acetyl-S-(phenyl)-L-cysteine (PMA) (Benzene)	0.60	9.1 hrs	
2-Thioxothiazolidine-4-carboxylic acid (TTCA) (Carbon Disulfide)	11.2	8 hrs	

Footnotes:

* Estimated from animal study. Multiple values indicate areas in which the literature lists multiple half-life values. N/A = “Not Available”

^ Assays were conducted for each of the seven major nicotine metabolites listed in eTable 1. In addition to the nicotine metabolites listed above, “Total Nicotine Equivalents” (TNE-2) was computed and included in statistical analyses as a reflection of systemic nicotine levels for participants. TNE-2 was calculated as the molar sum of cotinine and *trans*-3’-Hydroxycotinine. For arsenic and arsenic compounds, individual assays were conducted for each of the four listed compounds in eTable 1. The analysis used a summary variable, “Total Inorganic Arsenic”, representing the sum of the arsenous acid, arsenic acid, dimethylarsinic acid, and monomethylarsonic acid levels in each urine sample. As these are summary variables, TNE-2 and Total Inorganic Arsenic do not have listings for limits of detection.

© 2018 Goniewicz ML et al. *JAMA Network Open.*

eTable 2: Biomarkers of Exposure to Tobacco-Related Compounds and Other Chemicals, Among Current Never Users, e-Cigarette Only-Users, Dual Users, and Cigarette-Only Smokers (Normalized for Creatinine, Geometric Means, 95% Confidence Intervals), PATH Wave 1 (2013-2014) (n=5,105)

	Never users (1,655) (a)					Current e-cigarette-only users (n=247) (b)				
Biomarker	N	Geo Mean	95% CI Lower	95% CI Upper	% N Above LOD	N	Geo Mean	95% CI Lower	95% CI Upper	% N Above LOD
**Urinary Nicotine Metabolites (ng/mg creatinine)**										
Nicotine Equivalence (TNE2) (nmol/mg)	1,652	0.006[b]	0.005	0.007	99%	247	2.000[acd]	1.1	3.5	100%
trans-3'-Hydroxycotinine (HCTT)	1,641	6.9E-01[b]	6.00E-01	8.00E-01	98%	247	227.4[acd]	128.9	401.1	100%
Cotinine (COTT)	1,644	4.2E-01[b]	3.60E-01	4.90E-01	99%	247	124.3[acd]	68.9	224.4	100%
Nicotine (NICT)^	88	3.1E+01[b]	1.20E+01	8.00E+01	64%	179	423.6[acd]	306.7	584.9	93%
Cotinine N-oxide (COXT)^	88	1.4E+01[b]	6.80E+00	2.80E+01	99%	179	117.5[acd]	88.2	156.5	98%
Nicotine 1'-oxide (NOXT)^	88	1.1E+01[b]	4.20E+00	3.10E+01	76%	179	143.3[acd]	105.3	194.9	95%
Norcotinine (NCCT)^	88	4.3E+00[b]	2.30E+00	8.00E+00	95%	179	31.93[acd]	24.35	41.87	96%
Nornicotine (NNCT)^	88	3.4E+00[b]	1.80E+00	6.20E+00	35%	179	18.83[acd]	14.72	24.11	86%
**Minor Tobacco Alkaloids (ng/mg creatinine)**										
Anabasine (ANBT)^	88	0.605	0.347	1.053	29%	179	1.144[cd]	0.876	1.492	56%
Anatabine (ANTT)^	88	0.615	0.306	1.234	37%	179	0.886[cd]	0.658	1.192	46%
**Arsenic and Arsenic Compounds (ug/g creatinine)**										
Total Inorganic Arsenic	1,653	0.054	0.05	0.057	92%	247	0.053[d]	0.048	0.058	87%
**Tobacco Specific Nitrosamines (TSNAs) (pg/mg creatinine)**										
4-methylnitrosamino)-4-(3-pyridyl)-1-butanol (NNAL)	1,653	0.921[b]	0.819	1.035	51%	247	4.887[ad]	3.817	6.257	90%
N'-nitrosoanatabine (NAT)*	1,648	2.921[b]	2.739	3.114	1%	246	3.909[ad]	3.402	4.493	12%
N'-nitrosoanabasine (NAB)*	1,655	1.067[b]	1.003	1.135	2%	247	1.422[ad]	1.256	1.61	15%
N'-Nitrosonornicotine (NNN)*	1,647	1.923[b]	1.81	2.043	1%	241	3.471[ad]	3.033	3.972	34%
**Heavy Metals (ng/mg creatinine)**										
Beryllium (UBE)*	1,653	0.011[b]	0.01	0.011	3%	246	0.012[a]	0.011	0.014	9%
Cadmium (UCD)	1,652	0.149[b]	0.14	0.159	93%	246	0.193[ad]	0.165	0.225	92%
Cobalt (UCO)	1,653	0.564	0.537	0.591	100%	246	0.579	0.523	0.641	100%
Manganese (UMN)*	1,652	0.131	0.124	0.138	47%	246	0.14	0.124	0.158	47%
Lead (UPB)	1,653	0.351[b]	0.33	0.373	100%	246	0.432[a]	0.382	0.488	100%
Strontium (USR)	1,653	112.7	106.8	119	100%	246	118.9	101	140	100%

© 2018 Goniewicz ML et al. *JAMA Network Open.*

Thallium (UTL)	1,652	0.172	0.164	0.18	100%	246	0.169	0.153	0.188	99%
Uranium (UUR)	1,653	0.005[b]	0.005	0.006	85%	246	0.007[a]	0.006	0.008	90%

<u>eTable 2 – cont'</u>: Biomarkers of exposure to tobacco-related compounds and other chemicals, among current never users, e-cigarette only-users, dual users, and cigarette-only smokers (normalized for creatinine, geometric means, 95% confidence intervals), PATH Wave 1 (2013-2014) (n=5,105)

	Current dual users (n=792) (c)					Current cigarette-only smokers (n=2,411) (d)				
Biomarker	N	Geo Mean	95% CI Lower	95% CI Upper	% N Above LOD	N	Geo Mean	95% CI Lower	95% CI Upper	% N Above LOD
**Urinary Nicotine Metabolites (ng/mg creatinine)**										
Nicotine Equivalence (TNE2) (nmol/mg)	792	43.70[bd]	39.8	48.1	100%	2410	27.90[bc]	23.8	32.7	100%
trans-3'-Hydroxycotinine (HCTT)	791	4985.7[bd]	4533.5	5482.8	100%	2406	3182.3[bc]	2682.7	3773.2	100%
Cotinine (COTT)	792	2858.9 [bd]	2601.9	3141.2	100%	2410	1830.9[bc]	1577.4	2125.1	100%
Nicotine (NICT)^	779	1318.7[b]	1172.8	1482.8	98%	2299	1076.0[b]	967.7	1195.2	97%
Cotinine N-oxide (COXT)^	779	349.7[bd]	326.5	374.5	100%	2301	297.4[bc]	276.5	319.9	100%
Nicotine 1'-oxide (NOXT)^	779	387.4[bd]	352.6	425.6	99%	2301	326.4[bc]	302	352.7	98%
Norcotinine (NCCT)^	779	100.3[b]	93.4	107.7	100%	2301	90.51[b]	84.95	96.43	99%
Nornicotine (NNCT)^	779	67.94[b]	62.28	74.11	97%	2293	60.20[b]	56.04	64.67	96%
**Minor Tobacco Alkaloids (ng/mg creatinine)**										
Anabasine (ANBT)^	778	8.231[b]	7.429	9.12	96%	2301	7.799[b]	7.221	8.423	95%
Anatabine (ANTT)^	779	13.89[b]	12.35	15.61	96%	2298	13.02[b]	11.92	14.22	96%
**Arsenic and Arsenic Compounds (ug/g creatinine)**										
Total Inorganic Arsenic	790	0.047	0.045	0.05	78%	2409	0.048[b]	0.046	0.05	87%
**Tobacco Specific Nitrosamines (TSNAs) (pg/mg creatinine)**										
4-methylnitrosamino)-4-(3-pyridyl)-1-butanol (NNAL)	789	262.6[d]	240	287.3	100%	2408	203.5[bc]	181.7	227.9	99%
N'-nitrosoanatabine (NAT)*	776	126.9[d]	111.7	144.2	93%	2383	96.06[bc]	85.66	107.7	91%
N'-nitrosoanabasine (NAB)*	788	20.85[d]	18.62	23.34	92%	2402	15.67[bc]	14.12	17.39	88%
N'-Nitrosonornicotine (NNN)*	773	11.78	10.66	13.01	86%	2310	11.80[b]	10.84	12.85	85%
**Heavy Metals (ng/mg creatinine)**										
Beryllium (UBE)*	790	0.013	0.012	0.014	7%	2403	0.012	0.011	0.013	5%
Cadmium (UCD)	790	0.28	0.256	0.305	96%	2403	0.277[b]	0.259	0.297	97%
Cobalt (UCO)	790	0.6	0.566	0.637	100%	2403	0.542	0.524	0.56	100%

© 2018 Goniewicz ML et al. *JAMA Network Open.*

Manganese (UMN)*	790	0.153	0.143	0.165	41%	2402	0.137	0.13	0.143	41%
Lead (UPB)	790	0.5	0.475	0.526	100%	2403	0.479	0.462	0.496	100%
Strontium (USR)	787	130.5[d]	121.3	140.5	100%	2400	113.7[c]	107.3	120.6	100%
Thallium (UTL)	790	0.163	0.156	0.17	99%	2403	0.155	0.15	0.16	99%
Uranium (UUR)	790	0.008[d]	0.007	0.009	89%	2403	0.007[c]	0.006	0.008	88%

eTable 2 – cont': Biomarkers of exposure to tobacco-related compounds and other chemicals, among current never users, e-cigarette only-users, dual users, and cigarette-only smokers (normalized for creatinine, geometric means, 95% confidence intervals), PATH Wave 1 (2013-2014) (n=5,105)

	Never users (1,655) (a)					Current e-cigarette-only users (n=247) (b)				
Biomarker	N	Geo Mean	95% CI Lower	95% CI Upper	% N Above LOD	N	Geo Mean	95% CI Lower	95% CI Upper	% N Above LOD
**Polycyclic Aromatic Hydrocarbons (ng/mg creatinine)**										
1-Naphthol or 1-hydroxynaphthalene (1-NAP)	1,649	1.3992	1.28	1.529	100%	247	1.550[d]	1.216	1.975	100%
2-Naphthol or 2-hydroxynaphthalene (2-NAP)	1,652	4.6311	4.344	4.937	100%	246	5.287[d]	4.693	5.956	100%
3-Hydroxyfluorene (3-FLU)	1,655	0.064	0.06	0.068	99%	247	0.077[d]	0.068	0.086	98%
2-Hydroxyfluorene (2-FLU)	1,655	0.167	0.158	0.177	100%	247	0.199[d]	0.178	0.222	100%
1-Hydroxyphenanthrene (1-PHE)	1,655	0.106	0.101	0.112	100%	247	0.107[d]	0.096	0.12	99%
1-Hydroxypyrene (1-PYR)	1,655	0.128[b]	0.121	0.136	85%	247	0.161[ad]	0.143	0.181	89%
2-Hydroxyphenanthrene and 3-Hydroxyphenanthrene (2-3PHE)	1,655	0.129	0.123	0.135	100%	247	0.125[d]	0.113	0.139	98%
**Volatile Organic Compounds (VOC)[$] (ng/mg creatinine)**										
2-Methylhippuric acid (2MHA) (Xylene)	1,507	20.99	19.35	22.77	91%	210	27.77[d]	23.6	32.67	94%
3, 4-Methylhippuric acid (34MH) (Xylene)	1,569	154.9	145.8	164.5	100%	216	185.1[d]	159	215.6	100%
N-Acetyl-S-(2-carbamoylethyl)-L-cysteine (AAMA) (Acrylamide)	1,563	47.28	45.03	49.65	100%	215	56.05[d]	51.07	61.5	100%
N-Acetyl-S-(Nmethylcarbamoyl)-L-cysteine (AMCA) (N,N-Dimethylformamide/isocyantes)	1,571	105.1[b]	98.26	112.3	100%	216	153.8[ad]	136.1	173.8	100%
N-Acetyl-S-(benzyl)-L-cysteine (BMA) (Toluene)	1,571	6.314[b]	5.965	6.683	100%	216	6.985[a]	6.088	8.015	100%
N-Acetyl-S-(2-carboxyethyl)-L-cysteine (CEMA) (Acrolein)	1,517	98.14	93.89	102.6	99%	198	108.0[d]	95.93	121.6	100%
N-Acetyl-S-(1-cyano-2-hydroxyethyl)-L-cysteine (CYHA) (Acrylonitrile)*	1,571	1.870[b]	1.766	1.981	3%	216	2.431[ad]	2.114	2.794	14%
N-Acetyl-S-(2-cyanoethyl)-L-cysteine (CYMA) (Acrylonitrile)	1,571	1.315[b]	1.23	1.406	86%	216	3.959[ad]	3.002	5.219	94%
N-Acetyl-S-(3,4-dihydroxybutyl)-L-cysteine (DHBM) (1,3-Butadiene)	1,399	359	347.7	370.6	100%	189	360.2[d]	340.9	380.4	100%
N-Acetyl-S-(2-carbamoyl-2-hydroxyethyl)-L-cysteine (GAMA) (Acrylamide)	1,499	9.022	8.584	9.482	41%	203	9.924[d]	9.076	10.85	47%
N-Acetyl-S-(2-hydroxyethyl)-L-cysteine (HEMA) (Acrylonitrile, vinyl chloride, ethylene oxide)	1,426	0.955	0.893	1.02	51%	183	1.076[d]	0.945	1.224	53%
N-Acetyl-S-(2-hydroxypropyl)-L-cysteine (HPM2) (Propylene Oxide)	1,523	33.79	30.63	37.26	97%	214	34.45[d]	30.12	39.4	97%
N-Acetyl-S-(3-hydroxypropyl)-L-cysteine (HPMA) (Acrolein)	1,571	272.4	257	288.6	100%	212	314.8[d]	275.4	359.5	100%

© 2018 Goniewicz ML et al. *JAMA Network Open.*

N-Acetyl-S-(3-hydroxypropyl-1-methyl)-L-cysteine (HPMM) (Crotonaldehyde)	1,571	457.7	433.4	483.3	100%	216	442.8[d]	387.6	505.8	99%
N-Acetyl-S-(4-hydroxy-2-methyl-2-buten-1-yl)-L-cysteine (IPM3) (Isoprene)	1,555	3.378	3.155	3.617	89%	215	3.747[d]	3.247	4.323	91%
Mandelic acid (MADA)	1,382	131.7	126.6	136.9	99%	198	138.5[d]	119.5	159.8	97%
N-Acetyl-S-(4-hydroxy-2-buten-1-yl)-L-cysteine (MHB3) (1,3-Butadiene)	1,571	4.543	4.348	4.745	99%	216	4.308[d]	3.843	4.829	98%
Phenylglyoxylic acid (PGHA) (Ethylbenzene, styrene)	1,468	203.3	196.5	210.2	100%	203	223.7[d]	197.4	253.5	100%
N-Acetyl-S-(phenyl)-L-cysteine (PMA) (Benzene)	1,571	1.038	0.967	1.114	68%	216	1.007	0.9	1.125	69%
2-Thioxothiazolidine-4-carboxylic acid (TTCA) (Carbon Disulfide)	1,287	21.61	19.51	23.93	68%	186	19.36	15.99	23.43	68%

eTable 2 – cont': Biomarkers of exposure to tobacco-related compounds and other chemicals, among current never users, e-cigarette only-users, dual users, and cigarette-only smokers (normalized for creatinine, geometric means, 95% confidence intervals), PATH Wave 1 (2013-2014) (n=5,105)

	Current dual users (n=792) (c)					Current cigarette-only smokers (n=2,411) (d)				
Biomarker	N	Geo Mean	95% CI Lower	95% CI Upper	% N Above LOD	N	Geo Mean	95% CI Lower	95% CI Upper	% N Above LOD
**Polycyclic Aromatic Hydrocarbons (ng/mg creatinine)**										
1-Naphthol or 1-hydroxynaphthalene (1-NAP)	791	13.48[d]	11.91	15.25	100%	2411	11.11[bc]	10.18	12.12	100%
2-Naphthol or 2-hydroxynaphthalene (2-NAP)	791	14.79	14.01	15.61	100%	2411	13.91[b]	13.21	14.65	100%
3-Hydroxyfluorene (3-FLU)	792	0.630[d]	0.59	0.673	100%	2411	0.568[bc]	0.53	0.61	100%
2-Hydroxyfluorene (2-FLU)	792	1.141[d]	1.074	1.212	100%	2411	1.007[bc]	0.947	1.071	100%
1-Hydroxyphenanthrene (1-PHE)	792	0.200[d]	0.191	0.21	100%	2411	0.178[bc]	0.17	0.186	100%
1-Hydroxypyrene (1-PYR)	792	0.355[d]	0.339	0.373	99%	2411	0.303[bc]	0.287	0.321	97%
2-Hydroxyphenanthrene and 3-Hydroxyphenanthrene (2-3PHE)	792	0.316	0.299	0.333	100%	2411	0.303[b]	0.289	0.318	100%
**Volatile Organic Compounds (VOC)[$] (ng/mg creatinine)**										
2-Methylhippuric acid (2MHA) (Xylene)	736	109.3	101.6	117.6	99%	2274	98.769[b]	91.78	106.1	99%
3, 4-Methylhippuric acid (34MH) (Xylene)	769	758.4[d]	710.4	809.6	100%	2320	678.4[bc]	633.6	726.2	100%
N-Acetyl-S-(2-carbamoylethyl)-L-cysteine (AAMA) (Acrylamide)	764	144.0	136.4	151.9	100%	2303	136.4[b]	129.3	143.8	100%
N-Acetyl-S-(Nmethylcarbamoyl)-L-cysteine (AMCA) (N,N-Dimethylformamide/isocyantes)	767	550.3[d]	519.2	583.2	100%	2290	482.9[bc]	454	513.7	100%
N-Acetyl-S-(benzyl)-L-cysteine (BMA) (Toluene)	767	7.394[d]	6.836	7.998	100%	2309	6.696[c]	6.238	7.188	100%
N-Acetyl-S-(2-carboxyethyl)-L-cysteine (CEMA) (Acrolein)	739	302.0[d]	283.3	321.8	100%	2176	271.5[bc]	255.1	289	100%
N-Acetyl-S-(1-cyano-2-hydroxyethyl)-L-cysteine (CYHA) (Acrylonitrile)*	769	25.09	22.82	27.58	92%	2321	21.80[b]	20.05	23.69	88%
N-Acetyl-S-(2-cyanoethyl)-L-cysteine (CYMA) (Acrylonitrile)	769	146.2[d]	133.8	159.8	100%	2321	123.9[bc]	109.9	139.7	99%
N-Acetyl-S-(3,4-dihydroxybutyl)-L-cysteine (DHBM) (1,3-Butadiene)	732	532.7[d]	514.3	551.7	100%	2066	499.8[bc]	481.1	519.1	100%
N-Acetyl-S-(2-carbamoyl-2-hydroxyethyl)-L-cysteine (GAMA) (Acrylamide)	717	18.52	17.57	19.52	75%	2174	17.33[b]	16.49	18.21	74%

© 2018 Goniewicz ML et al. *JAMA Network Open.*

N-Acetyl-S-(2-hydroxyethyl)-L-cysteine (HEMA) (Acrylonitrile, vinyl chloride, ethylene oxide)	701	3.194[d]	2.936	3.475	88%	1988	2.744[bc]	2.545	2.958	83%
N-Acetyl-S-(2-hydroxypropyl)-L-cysteine (HPM2) (Propylene Oxide)	767	84.13[d]	78.12	90.6	100%	2301	71.10[bc]	67.59	74.8	99%
N-Acetyl-S-(3-hydroxypropyl)-L-cysteine (HPMA) (Acrolein)	767	1317.8[d]	1225	1417.7	100%	2284	1143.5[bc]	1064.3	1228.6	100%
N-Acetyl-S-(3-hydroxypropyl-1-methyl)-L-cysteine (HPMM) (Crotonaldehyde)	769	2707.7[d]	2515.8	2914.3	100%	2321	2359.3[bc]	2188.2	2543.8	100%
N-Acetyl-S-(4-hydroxy-2-methyl-2-buten-1-yl)-L-cysteine (IPM3) (Isoprene)	768	39.79[d]	36.33	43.56	99%	2316	33.50[bc]	30.69	36.56	99%
Mandelic acid (MADA)	734	287.8	274.4	301.9	99%	2112	279.3[b]	268.1	290.8	100%
N-Acetyl-S-(4-hydroxy-2-buten-1-yl)-L-cysteine (MHB3) (1,3-Butadiene)	769	31.92[d]	29.64	34.38	100%	2321	27.90[bc]	26.04	29.89	100%
Phenylglyoxylic acid (PGHA) (Ethylbenzene, styrene)	738	416.5[d]	399	434.8	100%	2192	375.8[bc]	360.3	391.8	100%
N-Acetyl-S-(phenyl)-L-cysteine (PMA) (Benzene)	769	1.071	1.017	1.127	64%	2321	1.090	1.035	1.147	69%
2-Thioxothiazolidine-4-carboxylic acid (TTCA) (Carbon Disulfide)	713	22.89	20.98	24.99	75%	2014	21.91	20.52	23.38	74%

Footnotes:

N reflects total number of cases included in estimate (excluding participants with results outside of quality control standards.) *Indicates analyte with >40% of all values falling below Limit of Detection (LOD) for select group. % LOD reflects proportion among cases included in estimate.

^ Detection rates for these metabolites only apply to participants who had urinary cotinine levels greater than 20 ng/L, which is reflected in the sample size for each biomarker.

Weighted estimates; geometric mean calculated by exp(mean of log transformed [biomarker value/creatinine value]). Some VOC laboratory results were outstanding at the time these analyses were conducted, so weighted estimates may not accurately reflect values in the target population.

Total Nicotine Equivalents (TNE2) calculated by taking molar sum of trans-3'-hydroxycotinine and cotinine divided by urinary creatinine. Geometric means expressed to four significant figures if under 1000; geometric means over 1000 expressed to five significant figures.

Red text denotes values with RSE>30% or denominator <50. Superscript letters denote statistically significant differences between tobacco use groups based on results from linear regression models adjusted for urinary creatinine, age, sex, ethnicity, education, SHS exposure, past 30 day marijuana use.($p<0.05$). Analyses comparing dual users and cigarette-only smokers also included CPD as a control variable. a=significantly different from never users, b=significantly different from e-cigarette-only users, c=significantly different from dual users, d=significantly different cigarette-only users.

[$] VOC information presented as Biomarker Name (Lab Code) (Parent Compound)

© 2018 Goniewicz ML et al. *JAMA Network Open.*

"Never users"= "No" to lifetime use of any tobacco product; " E-cigarette-only Users"=Reported "Yes" to current everyday/someday use of e-cigarettes, "No" to current use of all other tobacco products; " Dual Users"=Reported "Yes" to current everyday/someday use of tobacco cigarettes and e-cigarettes, "No" to current use of all other tobacco products; " Cigarette-only Smokers"=Reported "Yes" to current everyday/someday use of cigarettes, "No" to current use of all other tobacco products. All user groups reported no past three day use of Nicotine Replacement Therapies (NRT).

© 2018 Goniewicz ML et al. *JAMA Network Open.*

eMethods. Details on Model Building Procedures for Dual User Analysis and Sample Weighting

Dual User Models: All models examining dual users adjusted for CPD in addition to covariates listed in *Methods*. Models utilized multivariable linear regression modelling, using log transformed biomarker data as outcomes, and adjusted for urinary creatinine, demographic covariates, CPD, SHS exposure, past 30 day marijuana use, and a categorical variable indicating dual user status (1- everyday smoker/everyday e-cigarette user, 2- everyday e-cigarette user/somedays smoker, 3- everyday smoker/somedays e-cigarette user, 4 - somedays smoker/somedays e-cigarette user). Models were run using the highest exposure level (Group 1, everyday smokers/everyday e-cigarette users) and Group 2, "everyday smokers, somedays e-cigarette users" as the referent categories.

Weights: Since the biospecimen data represent a subsample of adults, specific urine weights were needed to account for potential differences between the full set of Adult Interview respondents and the set of adults with analyzed biospecimens. Weighting procedures are outlined in http://doi.org/10.3886/ICPSR36840.userguide.

© 2018 Goniewicz ML et al. *JAMA Network Open.*

eTable 3. Biomarkers of Exposure to Tobacco-Related Compounds and Other Chemicals, Among Everyday and Someday Users of e-Cigarettes and Cigarettes (normalized for creatinine, geometric means, 95% confidence intervals), PATH Wave 1 (2013-2014) (n=2,658)

Biomarker	Current e-cigarette-only users (n=247)									
	**Everyday users (n=147)**					**Somedays users (n=100)**				
	N	Geo Mean	95% CI Lower	95% CI Upper	% N Above LOD	N	Geo Mean	95% CI Lower	95% CI Upper	% N Above LOD
**Urinary Nicotine Metabolites (ng/mg creatinine)**										
Nicotine Equivalence (nmol/mg)	147	22.95*	16.35	32.23	100%	100	0.092#	0.049	0.171	100%
trans-3’-Hydroxycotinine (HCTT)	147	2547.7*	1803.7	3598.6	100%	100	10.89#	5.96	19.92	100%
Cotinine (COTT)	147	1480.5*	1035.6	2116.4	100%	100	5.513#	2.845	10.681	100%
Nicotine (NICT)^	143	684.4*	519.3	902.1	97%	36	64.77#	29.76	140.9	77%
Cotinine N-oxide (COXT)^	143	181.7*	142.5	231.6	100%	36	21.33#	10.59	43.01	89%
Nicotine 1’-oxide (NOXT)^	143	237.3*	182.9	307.9	99%	36	19.87#	9.221	42.8	77%
Norcotinine (NCCT)^	143	47.66*	37.37	60.79	99%	36	6.651#	3.535	12.51	85%
Nornicotine (NNCT)^	143	26.72*	21.31	33.51	93%	36	4.788#	2.864	8.006	58%
**Minor Tobacco Alkaloids (ng/mg creatinine)**										
Anabasine (ANBT)^	143	1.325*	0.997	1.758	59%	36	0.644	0.405	10.26	41%
Anatabine (ANTT)*^	143	1.001*	0.731	1.371	50%	36	0.55	0.305	9.898	32%
**Arsenic and Arsenic Compounds (ug/g creatinine)**										
Total Inorganic Arsenic	147	0.054*	0.048	0.061	86%	100	0.052	0.045	0.06	90%
**Tobacco Specific Nitrosamines (TSNAs) (pg/mg creatinine)**										
4-methylnitrosamino)-4-(3-pyridyl)-1-butanol (NNAL)	147	6.629*	5.083	8.645	93%	100	3.33	2.275	4.875	86%
N’-nitrosoanatabine (NAT)*	146	4.366*	3.725	5.117	15%	100	3.407	2.743	4.233	8%
N’-nitrosoanabasine (NAB)*	147	1.623*	1.395	1.89	21%	100	1.203#	0.992	1.459	8%
N'-Nitrosonornicotine (NNN)*	142	4.934*	4.123	5.904	54%	99	2.215#	1.86	2.637	9%
**Heavy Metals (ng/mg creatinine)**										
Beryllium (UBE)*	147	0.013	0.011	0.014	5%	99	0.012	0.01	0.014	14%
Cadmium (UCD)	147	0.258	0.219	0.305	97%	99	0.133	0.102	0.173	87%
Cobalt (UCO)	147	0.629*	0.56	0.705	100%	99	0.521	0.441	0.615	100%
Manganese (UMN)*	147	0.141	0.125	0.159	44%	99	0.139	0.112	0.172	53%
Lead (UPB)	147	0.514	0.433	0.609	99%	99	0.346#	0.289	0.414	100%
Strontium (USR)	147	144.6*	125.2	167.1	100%	99	92.73#	69.47	123.78	100%
Thallium (UTL)	147	0.179*	0.154	0.207	100%	99	0.158	0.14	0.179	99%

© 2018 Goniewicz ML et al. *JAMA Network Open.*

Uranium (UUR)	147	0.007	0.006	0.008	85%	99	0.007	0.006	0.008	96%

eTable 3 – cont': Biomarkers of exposure to tobacco-related compounds and other chemicals, among everyday and someday users of e-cigarettes and cigarettes (normalized for creatinine, geometric means, 95% confidence intervals), PATH Wave 1 (2013-2014) (n=2,658)

	**Current Cigarette-only Smokers (n=2,411)**									
Biomarker	**Everyday users (n=1,975)**					**Somedays users (n=436)**				
	N	Geo Mean	95% CI Lower	95% CI Upper	% N Above LOD	N	Geo Mean	95% CI Lower	95% CI Upper	% N Above LOD
**Urinary Nicotine Metabolites (ng/mg creatinine)**										
Nicotine Equivalence (nmol/mg)	1,974	45.94#	42.61	49.53	100%	436	2.082*#	1.29	3.4	100%
trans-3'-Hydroxycotinine (HCTT)	1,973	5189.6#	4767.2	5649.4	100%	433	243.8*#	149.1	398.7	100%
Cotinine (COTT)	1,974	3007.2#	2806.1	3222.6	100%	436	137.8*#	85.72	221.6	100%
Nicotine (NICT)^	1957	1438.6#	1312.3	1577.4	100%	342	144.5*#	106	197.1	81%
Cotinine N-oxide (COXT)^	1959	366.4#	345.6	388.5	100%	342	70.36*#	59.6	83.05	99%
Nicotine 1'-oxide (NOXT)^	1959	428.1#	400.9	457	100%	342	50.15*#	36.66	68.6	89%
Norcotinine (NCCT)^	1959	111.8#	106.3	117.5	100%	342	21.11*#	17.81	25.02	96%
Nornicotine (NNCT)^	1952	75.58#	71.09	80.36	100%	341	12.60*#	10.21	15.53	74%
**Minor Tobacco Alkaloids (ng/mg creatinine)**										
Anabasine (ANBT)^	1959	9.679#	9.015	10.39	99%	342	1.757*#	1.48	2.086	69%
Anatabine (ANTT)*^	1957	16.71#	15.4	18.14	99%	341	2.319*#	1.824	2.948	72%
**Arsenic and Arsenic Compounds (ug/g creatinine)**										
Total Inorganic Arsenic	1973	0.047#	0.045	0.049	86%	436	0.052*	0.047	0.056	91%
**Tobacco Specific Nitrosamines (TSNAs) (pg/mg creatinine)**										
4-methylnitrosamino)-4-(3-pyridyl)-1-butanol (NNAL)	1973	298.7#	276	323.2	100%	435	27.50*#	21.92	34.51	96%
N'-nitrosoanatabine (NAT)*	1950	140.0#	127.7	153.5	98%	433	13.56*#	10.86	16.93	54%
N'-nitrosoanabasine (NAB)*	1966	21.20#	19.34	23.24	95%	436	3.283*#	2.787	3.867	51%
N'-Nitrosonornicotine (NNN)*	1883	14.91#	13.83	16.08	94%	427	3.556*	3.176	3.982	41%
**Heavy Metals (ng/mg creatinine)**										
Beryllium (UBE)*	1967	0.012	0.012	0.013	5%	436	0.011	0.01	0.012	6%
Cadmium (UCD)	1967	0.313	0.294	0.334	97%	436	0.146*#	0.124	0.173	92%
Cobalt (UCO)	1967	0.547#	0.529	0.567	100%	436	0.513#	0.466	0.565	100%
Manganese (UMN)*	1966	0.14	0.133	0.148	41%	436	0.12	0.108	0.133	39%
Lead (UPB)	1967	0.496	0.476	0.517	100%	436	0.400#	0.375	0.426	100%

© 2018 Goniewicz ML et al. *JAMA Network Open.*

Strontium (USR)	1965	114.1#	107.1	121.5	100%	435	112.0#	99.91	125.5	100%
Thallium (UTL)	1967	0.154#	0.148	0.159	99%	436	0.162	0.152	0.173	100%
Uranium (UUR)	1967	0.007	0.006	0.008	89%	436	0.005	0.004	0.007	83%

eTable 3 – cont': Biomarkers of exposure to tobacco-related compounds and other chemicals, among everyday and someday users of e-cigarettes and cigarettes (normalized for creatinine, geometric means, 95% confidence intervals), PATH Wave 1 (2013-2014) (n=2,658)

	**Current e-cigarette-only users (n=247)**									
Biomarker	**Everyday users (n=147)**					**Somedays users (n=100)**				
	N	Geo Mean	95% CI Lower	95% CI Upper	% N Above LOD	N	Geo Mean	95% CI Lower	95% CI Upper	% N Above LOD
**Polycyclic Aromatic Hydrocarbons (ng/mg creatinine)**										
1-Naphthol or 1-hydroxynaphthalene (1-NAP)	147	1.640*	1.259	2.136	99%	100	1.445	0.93	2.243	100%
2-Naphthol or 2-hydroxynaphthalene (2-NAP)	146	5.080*	4.391	5.877	100%	100	5.556	4.678	6.596	100%
3-Hydroxyfluorene (3-FLU)	147	0.082*	0.07	0.096	96%	100	0.071	0.058	0.087	100%
2-Hydroxyfluorene (2-FLU)	147	0.214*	0.185	0.247	100%	100	0.181	0.155	0.21	100%
1-Hydroxyphenanthrene (1-PHE)	147	0.117*	0.102	0.134	100%	100	0.097	0.082	0.114	99%
1-Hydroxypyrene (1-PYR)	147	0.172*	0.147	0.201	88%	100	0.148	0.126	0.173	90%
2-Hydroxyphenanthrene and 3-Hydroxyphenanthrene (2-3PHE)	147	0.132*	0.118	0.149	98%	100	0.116	0.096	0.14	99%
**Volatile Organic Compounds (VOC)$ (ng/mg creatinine)**										
2-Methylhippuric acid (2MHA) (Xylene)	118	33.62*	26.56	42.54	92%	92	22.66	18.37	27.95	96%
3, 4-Methylhippuric acid (34MH) (Xylene)	123	206.5*	169.1	252.1	100%	93	164.2	129.9	207.5	100%
N-Acetyl-S-(2-carbamoylethyl)-L-cysteine (AAMA) (Acrylamide)	123	61.85*	54.23	70.53	100%	92	50.17	43.9	57.33	100%
N-Acetyl-S-(Nmethylcarbamoyl)-L-cysteine (AMCA) (N,N-Dimethylformamide/isocyantes)	123	203.8*	177.1	234.4	100%	93	112.6	92.2	137.6	100%
N-Acetyl-S-(benzyl)-L-cysteine (BMA) (Toluene)	123	7.088	5.812	8.644	100%	93	6.876	5.66	8.35	100%
N-Acetyl-S-(2-carboxyethyl)-L-cysteine (CEMA) (Acrolein)	113	120.6*	103.4	140.5	100%	85	95.78	81.39	112.8	100%
N-Acetyl-S-(1-cyano-2-hydroxyethyl)-L-cysteine (CYHA) (Acrylonitrile)*	123	2.630*	2.184	3.167	16%	93	2.226	1.768	2.802	12%
N-Acetyl-S-(2-cyanoethyl)-L-cysteine (CYMA) (Acrylonitrile)	123	4.804*	3.559	6.484	95%	93	3.194#	2.018	5.052	94%
N-Acetyl-S-(3,4-dihydroxybutyl)-L-cysteine (DHBM) (1,3-Butadiene)	106	384.2*	365.1	404.1	100%	83	337	305.4	371.8	100%
N-Acetyl-S-(2-carbamoyl-2-hydroxyethyl)-L-cysteine (GAMA) (Acrylamide)	115	10.70*	9.4	12.18	46%	88	9.153	8.062	10.39	45%
N-Acetyl-S-(2-hydroxyethyl)-L-cysteine (HEMA) (Acrylonitrile, vinyl chloride, ethylene oxide)	109	1.086*	0.925	1.274	47%	74	1.063	0.862	1.31	58%
N-Acetyl-S-(2-hydroxypropyl)-L-cysteine (HPM2) (Propylene Oxide)	122	38.96*	33.27	45.62	96%	92	30.05	25.17	35.9	100%

© 2018 Goniewicz ML et al. *JAMA Network Open.*

N-Acetyl-S-(3-hydroxypropyl)-L-cysteine (HPMA) (Acrolein)	121	365.4*	312.9	426.7	100%	91	267.2	218.2	327.1	100%
N-Acetyl-S-(3-hydroxypropyl-1-methyl)-L-cysteine (HPMM) (Crotonaldehyde)	123	441.3*	359.7	541.4	99%	93	444.4	387.5	509.7	100%
N-Acetyl-S-(4-hydroxy-2-methyl-2-buten-1-yl)-L-cysteine (IPM3) (Isoprene)	123	3.790*	3.225	4.452	88%	92	3.698	2.88	4.749	95%
Mandelic acid (MADA)	112	153.4*	121.2	194.2	97%	86	124.3	107	144.4	97%
N-Acetyl-S-(4-hydroxy-2-buten-1-yl)-L-cysteine (MHB3) (1,3-Butadiene)	123	4.344*	3.854	4.897	97%	93	4.27	3.6	5.064	99%
Phenylglyoxylic acid (PGHA) (Ethylbenzene, styrene)	113	237.0*	190.6	294.8	100%	90	210.8	184.1	241.1	100%
N-Acetyl-S-(phenyl)-L-cysteine (PMA) (Benzene)	123	1.067	0.938	1.214	68%	93	0.944	0.773	1.153	69%
2-Thioxothiazolidine-4-carboxylic acid (TTCA) (Carbon Disulfide)	107	19.51	15.24	24.98	67%	79	19.18	13.88	26.51	70%

eTable 3 – cont': Biomarkers of exposure to tobacco-related compounds and other chemicals, among everyday and someday users of e-cigarettes and cigarettes (normalized for creatinine, geometric means, 95% confidence intervals), PATH Wave 1 (2013-2014) (n=2,658)

Biomarker	Current Cigarette-only Smokers (n=2,411)									
	Everyday users (n=1,975)					Somedays users (n=436)				
	N	Geo Mean	95% CI Lower	95% CI Upper	% N Above LOD	N	Geo Mean	95% CI Lower	95% CI Upper	% N Above LOD
**Polycyclic Aromatic Hydrocarbons (ng/mg creatinine)**										
1-Naphthol or 1-hydroxynaphthalene (1-NAP)	1975	14.48#	13.32	15.74	100%	436	2.782*#	2.402	3.221	100%
2-Naphthol or 2-hydroxynaphthalene (2-NAP)	1975	15.93#	15.29	16.61	100%	436	6.849*#	6.265	7.486	100%
3-Hydroxyfluorene (3-FLU)	1975	0.713#	0.679	0.749	100%	436	0.174*#	0.154	0.197	100%
2-Hydroxyfluorene (2-FLU)	1975	1.234#	1.176	1.295	100%	436	0.348*#	0.318	0.381	100%
1-Hydroxyphenanthrene (1-PHE)	1975	0.193#	0.185	0.202	100%	436	0.117*	0.103	0.132	99%
1-Hydroxypyrene (1-PYR)	1975	0.336#	0.318	0.354	98%	436	0.178*	0.161	0.197	93%
2-Hydroxyphenanthrene and 3-Hydroxyphenanthrene (2-3PHE)	1975	0.339#	0.323	0.357	100%	436	0.169*#	0.156	0.184	100%
**Volatile Organic Compounds (VOC)[$] (ng/mg creatinine)**										
2-Methylhippuric acid (2MHA) (Xylene)	1861	117.7#	110.6	125.2	100%	413	40.04*#	34.61	46.32	96%
3, 4-Methylhippuric acid (34MH) (Xylene)	1899	811.2#	765.3	859.8	100%	421	272.6*#	241.1	308.2	100%
N-Acetyl-S-(2-carbamoylethyl)-L-cysteine (AAMA) (Acrylamide)	1885	153.3#	146.7	160.1	100%	418	74.64*#	66.07	84.33	100%
N-Acetyl-S-(Nmethylcarbamoyl)-L-cysteine (AMCA) (N,N-Dimethylformamide/isocyantes)	1873	572.4#	543	603.5	100%	417	203.6*#	186.4	222.3	100%
N-Acetyl-S-(benzyl)-L-cysteine (BMA) (Toluene)	1891	6.701	6.196	7.246	100%	418	6.674	5.596	7.96	100%
N-Acetyl-S-(2-carboxyethyl)-L-cysteine (CEMA) (Acrolein)	1782	310.7#	292.4	330.2	100%	394	137.8*#	125.5	151.2	100%
N-Acetyl-S-(1-cyano-2-hydroxyethyl)-L-cysteine (CYHA) (Acrylonitrile)*	1900	28.93#	26.99	31	96%	421	5.151*#	4.443	5.971	50%
N-Acetyl-S-(2-cyanoethyl)-L-cysteine (CYMA) (Acrylonitrile)	1900	179.3#	167	192.5	100%	421	18.81*#	14.33	24.68	97%
N-Acetyl-S-(3,4-dihydroxybutyl)-L-cysteine (DHBM) (1,3-Butadiene)	1683	528.5#	510.2	547.4	100%	383	379.5*#	353.6	407.2	100%
N-Acetyl-S-(2-carbamoyl-2-hydroxyethyl)-L-cysteine (GAMA) (Acrylamide)	1781	18.96#	18.2	19.75	75%	393	10.92*	9.72	12.28	51%

© 2018 Goniewicz ML et al. *JAMA Network Open.*

N-Acetyl-S-(2-hydroxyethyl)-L-cysteine (HEMA) (Acrylonitrile, vinyl chloride, ethylene oxide)	1629	3.172#	2.946	3.415	88%	359	1.317*#	1.129	1.537	62%
N-Acetyl-S-(2-hydroxypropyl)-L-cysteine (HPM2) (Propylene Oxide)	1884	79.74#	75.78	83.9	99%	417	39.64*	36.12	43.5	99%
N-Acetyl-S-(3-hydroxypropyl)-L-cysteine (HPMA) (Acrolein)	1873	1375.9#	1286.6	1471.2	100%	411	441.1*#	401.4	484.6	100%
N-Acetyl-S-(3-hydroxypropyl-1-methyl)-L-cysteine (HPMM) (Crotonaldehyde)	1900	2937.5#	2746.3	3141.9	100%	421	771.9*#	697.4	854.4	100%
N-Acetyl-S-(4-hydroxy-2-methyl-2-buten-1-yl)-L-cysteine (IPM3) (Isoprene)	1898	44.60#	41.47	47.97	100%	418	7.727*#	6.287	9.497	93%
Mandelic acid (MADA)	1730	305.9#	294.4	318	100%	382	175.6*#	162.1	190.1	99%
N-Acetyl-S-(4-hydroxy-2-buten-1-yl)-L-cysteine (MHB3) (1,3-Butadiene)	1900	34.36#	32.38	36.47	100%	421	9.649*#	8.222	11.32	100%
Phenylglyoxylic acid (PGHA) (Ethylbenzene, styrene)	1792	407.3#	390.7	424.5	100%	400	249.9*	227.1	275	100%
N-Acetyl-S-(phenyl)-L-cysteine (PMA) (Benzene)	1900	1.111	1.054	1.175	68%	421	0.986	0.88	1.104	70%
2-Thioxothiazolidine-4-carboxylic acid (TTCA) (Carbon Disulfide)	1649	22.26	20.75	23.87	74%	365	20.26	17.31	23.71	72%

Footnotes:

N reflects total number of cases included in estimate (excluding participants with results outside of quality control standards.) *Indicates analyte with >40% of all values falling below Limit of Detection (LOD) for select group. % LOD reflects proportion among cases included in estimate.

^ Detection rates for these metabolites only apply to participants who had urinary cotinine levels greater than 20 ng/L, which is reflected in the sample size for each biomarker

Weighted estimates, geometric mean calculated by exp(mean of log transformed [biomarker value/creatinine value]). Some VOC laboratory results were outstanding at the time these analyses were conducted, so weighted estimates may not accurately reflect values in the target population.

Total Nicotine Equivalents (TNE2) calculated by taking molar sum of trans-3'-hydroxycotinine and cotinine divided by urinary creatinine. Geometric means expressed to four significant figures if under 1000; geometric means over 1000 expressed to five significant figures.

Red text denotes values with RSE>30% or denominator <50

[$] VOC information presented as Biomarker Name (Lab Code) (Parent Compound)

* Denotes statistically significant difference when compared to daily cigarette-only smokers based on results from linear regression models adjusted for urinary creatinine, age, sex, ethnicity, education, SHS exposure, past 30 day marijuana use (p<0.05).

# Denotes statistically significant difference when compared to daily e-cigarette users based on results from linear regression models adjusted for urinary creatinine, age, sex, ethnicity, education, SHS exposure, past 30 day marijuana use, (p<0.05).

© 2018 Goniewicz ML et al. *JAMA Network Open.*

eTable 4. Biomarkers of Exposure to Tobacco-Related Compounds and Other Chemicals, Among Dual Users (normalized for creatinine, geometric means, 95% confidence intervals), PATH Wave 1 (2013-2014) (n=792)

	Everyday smokers/everyday e-cigarette users (n=90) (a)					Everyday e-cigarette users/somedays smoker (n=55) (b)				
Biomarker	N	Geo Mean	95% CI Lower	95% CI Upper	% N Above LOD	N	Geo Mean	95% CI Lower	95% CI Upper	% N Above LOD
**Urinary Nicotine Metabolites (ng/mg creatinine)**										
Nicotine Equivalence (TNE2) (nmol/mg)	90	66.40[d]	54	81.6	100%	55	35.10[d]	19.9	62	100%
trans-3'-Hydroxycotinine (HCTT)	90	7696.1[bd]	6343.2	9337.4	100%	55	3748.8[a]	2049.8	6855.9	100%
Cotinine (COTT)	90	4109.9[d]	3126	5403.6	100%	55	2443.9[d]	1420.1	4205.8	100%
Nicotine (NICT)^	90	1781.9[d]	1101.7	2881.9	100%	54	1210.9[d]	766.6	1912.9	100%
Cotinine N-oxide (COXT)^	90	439.0[d]	377.7	510.4	100%	54	313.5[d]	214.2	459.3	100%
Nicotine 1'-oxide (NOXT)^	90	557.3[d]	416.8	745.1	100%	54	449.2[d]	294.6	685.4	100%
Norcotinine (NCCT)^	90	138.8[d]	110.3	174.6	100%	54	80.91[d]	54	121.2	100%
Nornicotine (NNCT)^	90	87.61[d]	63.43	120.9	100%	54	50.95[d]	33.53	77.42	97%
**Minor Tobacco Alkaloids (ng/mg creatinine)**										
Anabasine (ANBT)^	89	9.450[d]	6.299	14.17	98%	54	3.727[c]	1.848	7.527	83%
Anatabine (ANTT)^	90	17.09[bd]	10.84	26.94	98%	54	4.845[ac]	2.148	10.93	82%
**Arsenic and Arsenic Compounds (ug/g creatinine)**										
Total Inorganic Arsenic	90	0.055	0.046	0.066	81%	55	0.048	0.04	0.059	95%
**Tobacco Specific Nitrosamines (TSNAs) (pg/mg creatinine)**										
4-methylnitrosamino)-4-(3-pyridyl)-1-butanol (NNAL)	90	354.9[bd]	266.9	472	100%	55	87.02[ac]	51.59	146.78	100%

© 2018 Goniewicz ML et al. *JAMA Network Open.*

N’-nitrosoanatabine (NAT)	89	163.0[bd]	105.8	251.3	98%	54	38.02[ac]	18.54	77.95	72%
N’-nitrosoanabasine (NAB)	90	25.14[bd]	16.37	38.59	95%	54	7.798[ac]	4.297	14.15	70%
N'-Nitrosonornicotine (NNN)	85	14.90[bd]	9.33	23.78	88%	54	6.768[ac]	4.802	9.54	78%
**Heavy Metals (ng/mg creatinine)**										
Beryllium (UBE)*	90	0.015[c]	0.011	0.019	2%	54	0.012	0.009	0.015	8%
Cadmium (UCD)	90	0.321[d]	0.259	0.398	96%	54	0.264	0.14	0.496	98%
Cobalt (UCO)	90	0.604	0.532	0.685	100%	54	0.691	0.467	1.021	100%
Manganese (UMN)*	90	0.164	0.132	0.204	32%	54	0.127	0.104	0.156	42%
Lead (UPB)	90	0.56	0.463	0.677	100%	54	0.473	0.403	0.555	100%
Strontium (USR)	90	139.9	119.5	163.9	100%	54	112.7	82.21	154.5	100%
Thallium (UTL)	90	0.153	0.137	0.171	100%	54	0.169	0.153	0.187	100%
Uranium (UUR)	90	0.008	0.006	0.011	85%	54	0.008	0.006	0.012	96%

eTable 4 – cont’: Biomarkers of exposure to tobacco-related compounds and other chemicals, among dual users (normalized for creatinine, geometric means, 95% confidence intervals), PATH Wave 1 (2013-2014) (n=792)

	Everyday smokers/somedays e-cigarette users (n=560) (c)					Somedays smokers/somedays e-cigarette users (n=87) (d)				
Biomarker	N	Geo Mean	95% CI Lower	95% CI Upper	% N Above LOD	N	Geo Mean	95% CI Lower	95% CI Upper	% N Above LOD
**Urinary Nicotine Metabolites (ng/mg creatinine)**										
Nicotine Equivalence (TNE2) (nmol/mg)	560	53.60[d]	49.5	58	100%	87	7.225[abc]	3.868	13.5	100%
trans-3’-Hydroxycotinine (HCTT)	560	5989.4[d]	5483.1	6542.6	100%	86	948.4[ac]	519.7	1730.8	100%
Cotinine (COTT)	560	3530.6[d]	3261.2	3822.1	100%	87	457.5[abc]	252.5	828.8	100%
Nicotine (NICT)^	558	1605.2[d]	1433.8	1797.2	99%	77	200.0[abc]	119.2	335.6	86%
Cotinine N-oxide (COXT)^	558	395.7[d]	368.9	424.3	100%	77	105.8[abc]	78.7	142.3	100%
Nicotine 1’-oxide (NOXT)^	558	449.0[d]	408.5	493.5	100%	77	65.40[abc]	42.25	101.2	91%
Norcotinine (NCCT)^	558	114.1[d]	106.5	122.3	100%	77	27.63[abc]	20.07	38.03	96%
Nornicotine (NNCT)^	558	80.46[d]	73.64	87.91	99%	77	16.05[abc]	11.71	22	82%
**Minor Tobacco Alkaloids (ng/mg creatinine)**										
Anabasine (ANBT)^	558	10.44[bd]	9.53	11.44	99%	77	2.021[ac]	1.486	2.747	79%
Anatabine (ANTT)^	558	18.43[bd]	16.7	20.35	99%	77	2.677[ac]	1.82	3.938	80%
**Arsenic and Arsenic Compounds (ug/g creatinine)**										
Total Inorganic Arsenic	559	0.046	0.044	0.049	75%	86	0.046	0.041	0.052	86%
**Tobacco Specific Nitrosamines (TSNAs) (pg/mg creatinine)**										

© 2018 Goniewicz ML et al. *JAMA Network Open.*

4-methylnitrosamino)-4-(3-pyridyl)-1-butanol (NNAL)	558	343.7[bd]	315.7	374.3	100%	86	59.96[ac]	40.68	88.39	98%
N'-nitrosoanatabine (NAT)	547	173.3[bd]	155.5	193.1	99%	86	25.66[ac]	16.92	38.92	67%
N'-nitrosoanabasine (NAB)	558	26.91[bd]	24.28	29.83	97%	86	5.544[ac]	3.947	7.787	66%
N'-Nitrosonornicotine (NNN)	548	13.90[bd]	12.54	15.42	93%	86	4.182[ac]	3.217	5.438	54%
**Heavy Metals (ng/mg creatinine)**										
Beryllium (UBE)*	559	0.013[a]	0.012	0.014	8%	87	0.012	0.01	0.014	5%
Cadmium (UCD)	559	0.297[d]	0.27	0.327	97%	87	0.159[ac]	0.127	0.2	90%
Cobalt (UCO)	559	0.597	0.554	0.643	100%	87	0.561	0.507	0.62	100%
Manganese (UMN)*	559	0.158	0.145	0.173	43%	87	0.129	0.111	0.151	36%
Lead (UPB)	559	0.502	0.472	0.535	100%	87	0.436	0.369	0.515	99%
Strontium (USR)	556	130.6	119.4	142.8	100%	87	132.5	118.2	148.5	100%
Thallium (UTL)	559	0.162	0.154	0.171	99%	87	0.178	0.157	0.202	99%
Uranium (UUR)	559	0.008	0.007	0.009	90%	87	0.007	0.006	0.009	88%

eTable 4 – cont': Biomarkers of exposure to tobacco-related compounds and other chemicals, among dual users (normalized for creatinine, geometric means, 95% confidence intervals), PATH Wave 1 (2013-2014) (n=792)

	**Everyday smokers/everyday e-cigarette users (n=90) (a)**					**Everyday e-cigarette users/somedays smoker (n=55) (b)**				
Biomarker	N	Geo Mean	95% CI Lower	95% CI Upper	% N Above LOD	N	Geo Mean	95% CI Lower	95% CI Upper	% N Above LOD
**Polycyclic Aromatic Hydrocarbons (ng/mg creatinine)**										
1-Naphthol or 1-hydroxynaphthalene (1-NAP)	90	15.51[d]	12.10	19.89	100%	55	6.703[c]	3.54	12.69	100%
2-Naphthol or 2-hydroxynaphthalene (2-NAP)	90	16.03[d]	13.67	18.79	100%	55	10.85[c]	7.197	16.34	100%
3-Hydroxyfluorene (3-FLU)	90	0.757[bd]	0.617	0.929	100%	55	0.319[ac]	0.207	0.493	100%
2-Hydroxyfluorene (2-FLU)	90	1.347[bd]	1.131	1.608	100%	55	0.626[ac]	0.425	0.921	100%
1-Hydroxyphenanthrene (1-PHE)	90	0.214[bd]	0.189	0.243	100%	55	0.147[ac]	0.118	0.183	100%
1-Hydroxypyrene (1-PYR)	90	0.368[bcd]	0.313	0.434	98%	55	0.217[ac]	0.173	0.271	100%
2-Hydroxyphenanthrene and 3-Hydroxyphenanthrene (2-3PHE)	90	0.353[bd]	0.296	0.422	100%	55	0.237[ac]	0.185	0.304	100%
**Volatile Organic Compounds (VOC)[$] (ng/mg creatinine)**										
2-Methylhippuric acid (2MHA) (Xylene)	84	135.7[bd]	111.8	164.7	100%	48	56.53[ac]	43.68	73.16	100%
3, 4-Methylhippuric acid (34MH) (Xylene)	85	833.3[bd]	705.8	983.9	100%	50	388.4[ac]	306.6	491.9	100%
N-Acetyl-S-(2-carbamoylethyl)-L-cysteine (AAMA) (Acrylamide)	83	166.7[bd]	144.9	191.6	100%	49	85.21[ac]	72.74	99.81	100%
N-Acetyl-S-(Nmethylcarbamoyl)-L-cysteine (AMCA) (N,N-Dimethylformamide/isocyanates)	84	578.6[bd]	493.9	677.7	100%	50	259.8[ac]	213.1	316.8	100%
N-Acetyl-S-(benzyl)-L-cysteine (BMA) (Toluene)	85	7.145	5.823	8.766	100%	50	6.910	5.004	9.541	100%

© 2018 Goniewicz ML et al. *JAMA Network Open.*

N-Acetyl-S-(2-carboxyethyl)-L-cysteine (CEMA) (Acrolein)	80	316.9[bd]	267.6	375.4	100%	48	182.0[ac]	149.7	221.2	100%
N-Acetyl-S-(1-cyano-2-hydroxyethyl)-L-cysteine (CYHA) (Acrylonitrile)	85	26.38[bcd]	21.37	32.55	94%	50	9.114[ac]	6.224	13.35	70%
N-Acetyl-S-(2-cyanoethyl)-L-cysteine (CYMA) (Acrylonitrile)	85	180.5[bd]	147.1	221.6	100%	50	39.39[ac]	24.55	63.19	100%
N-Acetyl-S-(3,4-dihydroxybutyl)-L-cysteine (DHBM) (1,3-Butadiene)	76	527.2	475.3	584.7	100%	47	413.0[c]	366.3	465.8	100%
N-Acetyl-S-(2-carbamoyl-2-hydroxyethyl)-L-cysteine (GAMA) (Acrylamide)	83	19.73[bd]	17.32	22.46	71%	44	12.19[ac]	10.37	14.33	77%
N-Acetyl-S-(2-hydroxyethyl)-L-cysteine (HEMA) (Acrylonitrile, vinyl chloride, ethylene oxide)	78	3.397[bd]	2.768	4.17	89%	41	1.521[ac]	1.057	2.185	78%
N-Acetyl-S-(2-hydroxypropyl)-L-cysteine (HPM2) (Propylene Oxide)	85	89.24	70.88	112.4	100%	50	59.06[c]	46.53	74.97	100%
N-Acetyl-S-(3-hydroxypropyl)-L-cysteine (HPMA) (Acrolein)	85	1487.9[bd]	1246	1776.8	100%	50	618.1[ac]	519.5	735.5	100%
N-Acetyl-S-(3-hydroxypropyl-1-methyl)-L-cysteine (HPMM) (Crotonaldehyde)	84	2947.1[bcd]	2495.2	3480.9	100%	50	1091.9[ac]	892.3	1336.2	100%
N-Acetyl-S-(4-hydroxy-2-methyl-2-buten-1-yl)-L-cysteine (IPM3) (Isoprene)	85	44.58[bd]	35.48	56.02	100%	50	12.83[ac]	9.278	17.75	97%
Mandelic acid (MADA)	76	289.7[bd]	249.5	336.4	99%	47	176.5[ac]	147.1	211.6	99%
N-Acetyl-S-(4-hydroxy-2-buten-1-yl)-L-cysteine (MHB3) (1,3-Butadiene)	85	32.71[bcd]	27.44	38.99	100%	50	13.45[ac]	10.48	17.26	100%
Phenylglyoxylic acid (PGHA) (Ethylbenzene, styrene)	81	404.6 [bcd]	360.3	454.3	100%	50	289.6[ac]	256.7	326.6	100%
N-Acetyl-S-(phenyl)-L-cysteine (PMA) (Benzene)	85	1.147	0.985	1.336	60%	50	0.886	0.72	1.092	67%
2-Thioxothiazolidine-4-carboxylic acid (TTCA) (Carbon Disulfide)	76	25.76	20.36	32.61	72%	44	20.02	14.2	28.23	73%

<u>eTable 4 – cont':</u> Biomarkers of exposure to tobacco-related compounds and other chemicals, among dual users (normalized for creatinine, geometric means, 95% confidence intervals), PATH Wave 1 (2013-2014) (n=792)

	**Everyday smokers/somedays e-cigarette users (n=560) (c)**					**Somedays smokers/somedays e-cigarette users (n=87) (d)**				
Biomarker	N	Geo Mean	95% CI Lower	95% CI Upper	% N Above LOD	N	Geo Mean	95% CI Lower	95% CI Upper	% N Above LOD
**Polycyclic Aromatic Hydrocarbons (ng/mg creatinine)**										
1-Naphthol or 1-hydroxynaphthalene (1-NAP)	559	16.47[bd]	14.5	18.71	100%	87	4.618[ac]	2.96	7.204	100%
2-Naphthol or 2-hydroxynaphthalene (2-NAP)	559	16.35[bd]	15.5	17.24	100%	87	8.299[ac]	6.917	9.958	100%
3-Hydroxyfluorene (3-FLU)	560	0.752[bd]	0.701	0.807	100%	87	0.237[ac]	0.184	0.306	99%
2-Hydroxyfluorene (2-FLU)	560	1.326[bd]	1.242	1.416	100%	87	0.500[ac]	0.401	0.622	100%
1-Hydroxyphenanthrene (1-PHE)	560	0.215[bd]	0.203	0.227	100%	87	0.142[ac]	0.121	0.166	100%
1-Hydroxypyrene (1-PYR)	560	0.395[abd]	0.374	0.418	99%	87	0.228[ac]	0.193	0.269	96%
2-Hydroxyphenanthrene and 3-Hydroxyphenanthrene (2-3PHE)	560	0.345[bd]	0.324	0.368	100%	87	0.182[ac]	0.157	0.21	100%
**Volatile Organic Compounds (VOC)[$] (ng/mg creatinine)**										
2-Methylhippuric acid (2MHA) (Xylene)	521	125.8[bd]	115.4	137.1	99%	83	47.87[ac]	37.58	60.97	99%
3, 4-Methylhippuric acid (34MH) (Xylene)	548	894.9[bd]	831.4	963.2	100%	86	321.3[ac]	254.4	405.7	100%
N-Acetyl-S-(2-carbamoylethyl)-L-cysteine (AAMA) (Acrylamide)	546	157.3[bd]	147.8	167.3	100%	86	89.79[ac]	77.16	104.5	100%

© 2018 Goniewicz ML et al. *JAMA Network Open.*

N-Acetyl-S-(Nmethylcarbamoyl)-L-cysteine (AMCA) (N,N-Dimethylformamide/isocyanates)	547	641.7[bd]	600.4	685.9	100%	86	277.7[ac]	228.4	337.6	100%
N-Acetyl-S-(benzyl)-L-cysteine (BMA) (Toluene)	547	7.459	6.826	8.151	100%	85	7.494	6.032	9.31	100%
N-Acetyl-S-(2-carboxyethyl)-L-cysteine (CEMA) (Acrolein)	527	342.0[bd]	318.5	367.3	100%	84	166.0[ac]	136.2	202.2	100%
N-Acetyl-S-(1-cyano-2-hydroxyethyl)-L-cysteine (CYHA) (Acrylonitrile)	548	32.30[abd]	29.26	35.66	97%	86	7.451[ac]	5.354	10.37	65%
N-Acetyl-S-(2-cyanoethyl)-L-cysteine (CYMA) (Acrylonitrile)	548	194.9[bd]	177.7	213.8	100%	86	33.99[ac]	22.84	50.6	99%
N-Acetyl-S-(3,4-dihydroxybutyl)-L-cysteine (DHBM) (1,3-Butadiene)	527	561.4[bd]	537.3	586.6	100%	82	430.6[c]	399.6	464.1	100%
N-Acetyl-S-(2-carbamoyl-2-hydroxyethyl)-L-cysteine (GAMA) (Acrylamide)	509	19.81[bd]	18.6	21.09	76%	81	13.45[ac]	11.93	15.16	67%
N-Acetyl-S-(2-hydroxyethyl)-L-cysteine (HEMA) (Acrylonitrile, vinyl chloride, ethylene oxide)	499	3.674[bd]	3.314	4.073	90%	83	1.707[ac]	1.342	2.172	71%
N-Acetyl-S-(2-hydroxypropyl)-L-cysteine (HPM2) (Propylene Oxide)	546	92.23[bd]	84.56	100.6	100%	86	51.47[c]	41.59	63.71	99%
N-Acetyl-S-(3-hydroxypropyl)-L-cysteine (HPMA) (Acrolein)	546	1563.8[bd]	1436.6	1702.2	100%	86	551.6[ac]	449.6	676.6	100%
N-Acetyl-S-(3-hydroxypropyl-1-methyl)-L-cysteine (HPMM) (Crotonaldehyde)	537	3233.1[abd]	2975.1	3513.4	100%	86	1063.7[ac]	846.4	1336.8	100%
N-Acetyl-S-(4-hydroxy-2-methyl-2-buten-1-yl)-L-cysteine (IPM3) (Isoprene)	547	51.27[bd]	46.86	56.09	99%	86	11.78[ac]	8.454	16.4	93%
Mandelic acid (MADA)	530	316.7[bd]	298.2	336.4	99%	81	190.8[ac]	164.5	221.2	98%
N-Acetyl-S-(4-hydroxy-2-buten-1-yl)-L-cysteine (MHB3) (1,3-Butadiene)	548	39.52[abd]	36.46	42.84	99%	86	11.70[ac]	9.037	15.15	100%
Phenylglyoxylic acid (PGHA) (Ethylbenzene, styrene)	524	457.9 [abd]	435.1	481.8	100%	83	280.4[ac]	245.3	320.5	100%
N-Acetyl-S-(phenyl)-L-cysteine (PMA) (Benzene)	548	1.074	1.007	1.146	63%	86	1.088	0.928	1.275	71%
2-Thioxothiazolidine-4-carboxylic acid (TTCA) (Carbon Disulfide)	510	22.97	20.93	25.19	77%	83	21.27	16.46	27.49	67%

Footnotes:

N reflects total number of cases included in estimate (excluding participants with results outside of quality control standards.) *Indicates analyte with >40% of all values falling below Limit of Detection (LOD) for select group. % LOD reflects proportion among cases included in estimate.

^ Detection rates for these metabolites only apply to participants who had urinary cotinine levels greater than 20 ng/L, which is reflected in the sample size for each biomarker

Weighted estimates, geometric mean calculated by exp(mean of log transformed [biomarker value/creatinine value]). Some VOC laboratory results were outstanding at the time these analyses were conducted, so weighted estimates may not accurately reflect values in the target population. Total Nicotine Equivalents (TNE2) calculated by taking molar sum of trans-3'-hydroxycotinine and cotinine divided by urinary creatinine. Geometric means expressed to four significant figures if under 1000; geometric means over 1000 expressed to five significant figures.

Red text denotes values with RSE>30% or denominator <50. Superscript letters denote statistically significant differences between dual user groups based on results from linear regression models adjusted for urinary creatinine, CPD, age, sex, ethnicity, education, SHS exposure, past 30 day marijuana use, and tobacco use status (p<0.05).

© 2018 Goniewicz ML et al. *JAMA Network Open.*

[$] VOC information presented as Biomarker Name (Lab Code) (Parent Compound)

© 2018 Goniewicz ML et al. *JAMA Network Open.*

eReferences

1. Li Z, Romanoff L, Bartell S, et al. Excretion Profiles and Half-Lives of Ten Urinary Polycyclic Aromatic Hydrocarbon Metabolites after Dietary Exposure. *Chemical research in toxicology*. 2012;25(7):1452-1461.
2. Bernert JT, Harmon TL, Sosnoff CS, McGuffey JE. Use of continine immunoassay test strips for preclassifying urine samples from smokers and nonsmokers prior to analysis by LC-MS-MS. *Journal of analytical toxicology*. 2005;29(8):814-818.
3. Wei B, Feng J, Rehmani IJ, et al. A high-throughput robotic sample preparation system and HPLC-MS/MS for measuring urinary anatabine, anabasine, nicotine and major nicotine metabolites. *Clinica chimica acta; international journal of clinical chemistry*. 2014;436:290-297.
4. Caldwell KL, Jones RL, Verdon CP, Jarrett JM, Caudill SP, Osterloh JD. Levels of urinary total and speciated arsenic in the US population: National Health and Nutrition Examination Survey 2003-2004. *Journal of exposure science & environmental epidemiology*. 2009;19(1):59-68.
5. Verdon CP, Caldwell KL, Fresquez MR, Jones RL. Determination of seven arsenic compounds in urine by HPLC-ICP-DRC-MS: a CDC population biomonitoring method. *Analytical and bioanalytical chemistry*. 2009;393(3):939-947.
6. Goniewicz ML, Havel CM, Peng MW, et al. Elimination kinetics of the tobacco-specific biomarker and lung carcinogen 4-(methylnitrosamino)-1-(3-pyridyl)-1-butanol. *Cancer epidemiology, biomarkers & prevention : a publication of the American Association for Cancer Research, cosponsored by the American Society of Preventive Oncology*. 2009;18(12):3421-3425.
7. Xia B, Xia Y, Wong J, et al. Quantitative analysis of five tobacco-specific N-nitrosamines in urine by liquid chromatography-atmospheric pressure ionization tandem mass spectrometry. *Biomedical chromatography : BMC*. 2014;28(3):375-384.
8. Caldwell KL, Hartel J, Jarrett J, Jones RL. Inductively coupled plasma mass spectrometry to measure multiple toxic elements in urine in NHANES 1999-2000. *Atomic Spectroscopy*. 2005;26(1):1-7.
9. Jarrett JM, Xiao G, Caldwell KL, Henahan D, Shakirova G, Jones RL. Eliminating molybdenum oxide interference in urine cadmium biomonitoring using ICP-DRC-MS. *Journal of Analytical Atomic Spectrometry*. 2008;23(7):962.
10. St Helen G, Goniewicz ML, Dempsey D, Wilson M, Jacob P, 3rd, Benowitz NL. Exposure and kinetics of polycyclic aromatic hydrocarbons (PAHs) in cigarette smokers. *Chemical research in toxicology*. 2012;25(4):952-964.
11. Wang Y, Meng L, Pittman EN, et al. Quantification of urinary mono-hydroxylated metabolites of polycyclic aromatic hydrocarbons by on-line solid phase extraction-high performance liquid chromatography-tandem mass spectrometry. *Analytical and bioanalytical chemistry*. 2017;409(4):931-937.
12. Alwis KU, Bailey TL, Patel D, Wang L, Blount BC. Measuring urinary N-acetyl-S-(4-hydroxy-2-methyl-2-buten-1-yl)-L-cysteine (IPMA3) as a potential biomarker of isoprene exposure. *Analitica chimica acta*. 2016;941:61-66.
13. Alwis KU, Blount BC, Britt AS, Patel D, Ashley DL. Simultaneous analysis of 28 urinary VOC metabolites using ultra high performance liquid chromatography coupled with electrospray ionization tandem mass spectrometry (UPLC-ESI/MSMS). *Analytica chimica acta*. 2012;750:152.

© 2018 Goniewicz ML et al. *JAMA Network Open.*
